# Piezo1-mediated mechano-energetics regulate CAR T cell function

**DOI:** 10.21203/rs.3.rs-7776704/v1

**Published:** 2025-10-23

**Authors:** Ngoc Luu, Rui Li, Yifei Fang, Huishu Wang, Yujing Song, Ruiqi Chen, Xiangyi Fang, Junru Liao, Tracy Chen, Andre Kelly, Alexander A. Shestov, Katsuo Kurabayashi, Roddy O’Connor, Louis Hodgson, Saba Ghassemi, Weiqiang Chen

**Affiliations:** 1 Department of Biomedical Engineering, Tandon School of Engineering, New York University, Brooklyn, NY 11201, USA.; 2 Department of Mechanical and Aerospace Engineering, Tandon School of Engineering, New York University, Brooklyn, NY 11201, USA.; 3 Department of Chemical and Biomolecular Engineering, Tandon School of Engineering, New York University, Brooklyn, NY 11201, USA.; 4 Center for Cellular Immunotherapies, Perelman School of Medicine at the University of Pennsylvania, Philadelphia, PA 19104, USA.; 5 Department of Pathology and Laboratory Medicine, Perelman School of Medicine at the University of Pennsylvania, Philadelphia, PA 19104, USA.; 6 Department of Molecular Pharmacology, Albert Einstein College of Medicine, Bronx, NY 10461, USA.; 7 Gruss-Lipper Biophotonics Center, Albert Einstein College of Medicine, Bronx, NY 10461, USA.; 8 Perlmutter Cancer Center, NYU Grossman School of Medicine, New York, NY 10016, USA.

## Abstract

CAR T cell cytotoxicity requires generating immense mechanical force, but the energetic costs of this process remain poorly defined. While metabolic reprogramming fuels effector function, its mechanistic connection to mechanotransduction remains unclear. By directly measuring the synaptic force and mechanical energy of single CAR T cells and linking them to their metabolic state, we proved that the mechano-energetic efficiency is a fundamental determinant of cytotoxic potency. We discovered that the mechanosensitive ion channel Piezo1 couples cytoskeletal dynamics to metabolic rewiring via Ca^2+^-Wnt-Rac1 signaling. Disrupting Piezo1 cripples glycolytic and mitochondrial ATP production, causing energetic stress and impaired cytotoxicity. Notably, Piezo1 activity follows a Goldilocks principle: intermediate level maximizes activation and cytotoxicity, whereas either hypoactive or hyperactive Piezo1 states impair mechano-metabolic fitness and drive dysfunction in patient and exhausted CAR T cells. Our work establishes mechano-metabolic coupling as a core regulator of CAR T cell fitness and pinpoints Piezo1 tuning as a new strategy to enhance cancer immunotherapy.

Chimeric antigen receptor (CAR) T cell therapy has transformed the treatment of hematological malignancies by redirecting patients’ own T cells to eliminate cancer, yet despite its remarkable success, responses remain variable and relapse is frequent^1–3^. These challenges highlight the importance of understanding how CAR T cells adapt to the hostile tumor microenvironment, where they must integrate biochemical, metabolic, and physical cues^4^. T cell activation require dynamic mechanical allostasis, with actomyosin-driven contractility and retrograde actin flow to stabilize the immunological synapse^5^. This mechanobiological process demands energetic investment^6^, consume ATP and trigger metabolic rewiring^7^. During early activation, T cells shift from oxidative phosphorylation to aerobic glycolysis, supporting biosynthesis, rapid proliferation, and effector function^8^. However, patient-derived CAR T cell products frequently exhibit functional impairments that have been associated with altered metabolic fitness and mechanotransduction^9^. Such defects, compounded by variability in T cell quality, manufacturing processes, and in vivo exhaustion, collectively shape CAR T cell performance^10^. While the role of mechanics in TCR signaling has been extensively studied^11, 12^, CAR T cells exhibit distinct behaviors - faster killing and altered synapse morphology^13^, suggesting unique mechano-sensing mechanisms. Heterogeneity in CAR design and patient-specific T cell states further diversify these processes, yet the mechanobiological underpinnings of CAR T cell function remain underexplored.

Although mechanical signaling and metabolic rewiring have each been separately characterized, the systematic connection between mechanical inputs, metabolic and functional outputs in CAR T cells remains underexplored. Given the high energetic cost of actin remodeling, migration, and synapse stabilization, cytoskeletal dynamics and metabolic fitness must be tightly coupled^14^. Indeed, cells with poor metabolism show force dysregulation and loss of cytotoxic function^15^, while cells with strong metabolism but poor mechanical signaling may fail in vivo^16^. In the tumor microenvironment, where CAR T cells encounter both mechanical and metabolic stress, this coupling likely dictates persistence versus exhaustion^17, 18^.

Piezo1, a mechanosensitive cation channel, has emerged as a central candidate linking mechanical inputs to downstream signaling^19–22^. Piezo1 mediates calcium (Ca^2+^) influx that activates NFAT, reshapes the actin cytoskeleton via GTPases, and rewires metabolism through glycolysis, oxidative phosphorylation, and mTOR^23–29^. Yet its role in T cells remains controversial. Some studies suggested that Piezo1 is essential for antigen recognition, cytoskeletal remodeling, and cytotoxicity, positioning it as a core regulator of T cell immunity^30–32^. Others demonstrated that Piezo1 is dispensable for T cell function, where genetic deletion increase traction force and cytotoxic function^33–35^. This paradox underscores the complexity of Piezo1 signaling and highlights the need for context-specific mechanistic studies. Moreover, how Piezo1 links cytoskeletal, metabolic, and mechanotransduction in CAR T cells remains unclear^31, 36^.

Here, using high-resolution single-cell biosensing platforms, we track synaptic force, actomyosin remodeling, and mechanical energy in single CAR T cells upon antigen stimulation. We uncover how Piezo1-mediated mechanotransduction integrates with metabolic reprogramming to shape the mechano-energetic landscape and functional heterogeneity across donors. Our findings establish a mechano-metabolic framework that informs strategies to engineer next-generation CAR T cell therapies with improved mechanical fitness and metabolic resilience.

## Results

### Driving machinery in CAR T cell activation and cytotoxicity: Force matters.

**Extended Data Fig. 1.**
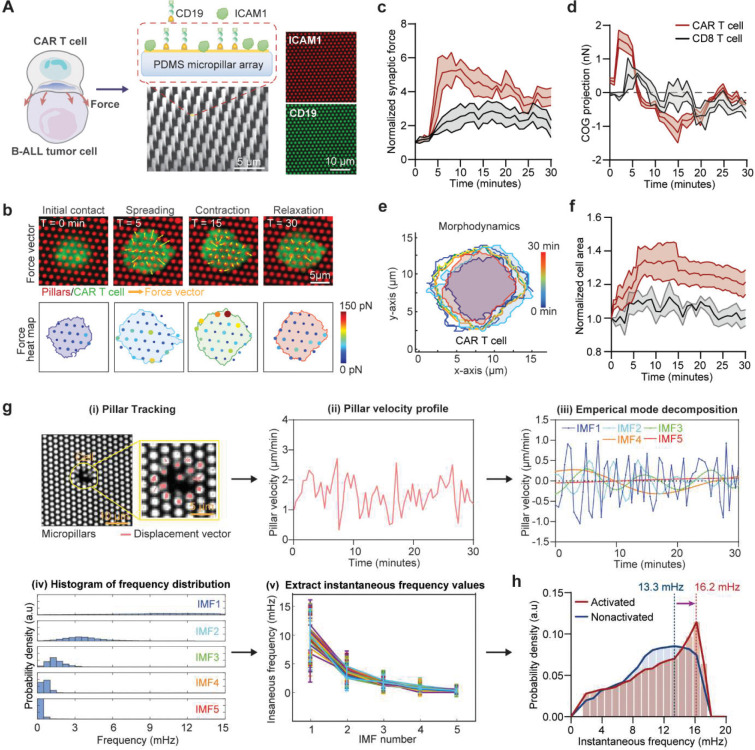
CAR T cell synaptic force measurement and instantaneous frequency spectrum analysis. (**a**) Schematic (left), SEM image (bottom middle), and immunofluorescent images (right) showing the microengineered PDMS micropillar array functionalized with CD19 and ICAM-1 to mimic the CAR–CD19 synapse. Schematic (left part), Created in BioRender. (**b**) Representative time-lapse force response of a CellTrace Violet-labeled CAR T cell (green) seeded on CD19-functionalized micropillars (red). Top: Force vector maps at key timepoints (T = 0, 5, 15, 30 min); bottom: corresponding force magnitude heatmaps. Force maps were generated using a custom MATLAB pipeline and Cellogram based on micropillar displacements within the segmented cell boundary. (**c**) Quantification of normalized synaptic force over 30 minutes in CAR T cells (red) vs. CD8^+^ T cells (black) stimulated on anti-CD3ε/anti-CD28/ICAM-1/fibrinogen-coated micropillars (n = 20 cells for CAR T and n = 10 cells for CD8+ T cells). (**d**) Directionality of force measured via projection of displacement vectors onto each cell’s center of gravity (COG). Positive COG projection indicates outward force (spreading); negative indicates inward (contraction) (n = 10 cells per group). (**e**) Representative cell morphodynamics over time for CAR T cells (n = 20 cells), calculated from binary segmentation of CellTrace Violet-labeled cells across time-lapse images. Boundaries from each timepoint are overlaid and color-coded by time (0–30 min). (**f**) Normalized change in cell area for CAR T and CD8^+^ T cells over time (n = 20 cells for CAR T and n = 10 cells for CD8+ T cells). Cell area was extracted from segmented images as described in (e). In c–f, thick lines represent the mean, and shaded areas denote s.e.m. All data were derived from two independent experiments. (**g**) Framework of instantaneous frequency spectrum analysis of synaptic force dynamics based on Hilbert-Huang transform methodology: (i) Tracking of individual pillar (white dots) displacement over time (red arrow). (ii) Calculation of micropillar velocity from displacement over 30 minutes. (iii) Empirical mode decomposition (EMD) of the velocity signal to decompose the original time series into Intrinsic Mode Functions (IMF). This study used 5 IMFs, determined by a specific end condition: the variance of the leftover signal (calculated by subtracting the sum of IMF1 through IMF5 from the original signal) needed to be below 5% of the original signal’s variance. (iv) Application of Hilbert-Hang transforms to derive the frequency distribution. Each histogram shows frequency distribution of input signals from each IMF. (v) Plotting shows instantaneous frequency values extracted from the frequency spectra. (**h**) IMF1 frequency spectrum were used to differentiate the distinct force dynamics of activated and nonactivated CAR T cells.

**Figure 1.**
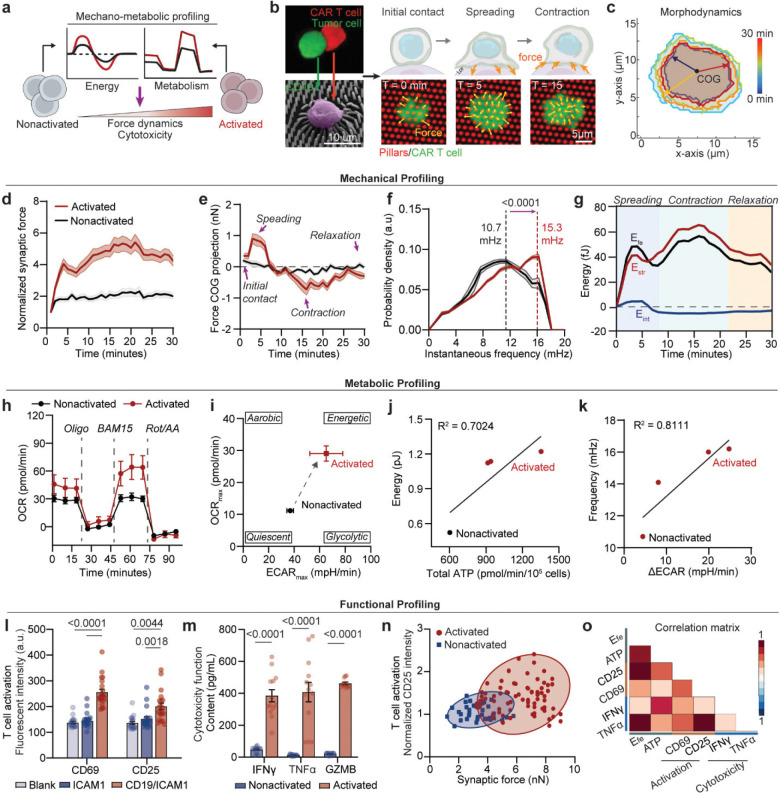
Mapping the synaptic force and mechanoenergetic landscape of CAR T cells upon activation. (**a**) Schematic outlining the single-cell micromechanical measurement of synaptic force, energy and force instantaneous frequency spectrum output signals to assess mechanical behaviors and function of single CAR T cells. Schematic, Created in BioRender. (**b**) Left panel: Representative immunofluorescence image (top) illustrating interaction between a CAR T cell and a B-ALL cell and SEM image (bottom) illustrating a CAR T cell applying synaptic force on a CD19/ICAM-1-functioned PDMS micropillar array. Right panel: A schematic (right top panel) illustrating the spatiotemporal morphodynamics and representative immunofluorescence images showing synaptic force response (right bottom panel) of a CAR T cell (green, labeled with CellTrace Violet) during initial contact, spreading, and contraction phases upon CD19 activation on a CD19/ICAM-1-functionalized micropillar array (red). Pseudo blue color is used in the SEM image to highlight the CAR T cell. (**c**) Morphodynamic tracking of a CAR T cell based on time-lapse imaging, binary thresholding, and morphological segmentation. (**d**) Magnitude of the synaptic force, (**e**) direction of the synaptic force based on center of gravity (COG) projection of cell, (**f**) instantaneous frequency distributions of force dynamic responses, and (**g**) change in free energy ($E_{\mathrm{free}}$) of CAR T cells, composed of strain energy ($E_{\mathrm{strain}}$) and interfacial energy ($E_{\mathrm{int}}$), from 0 to 30 min upon CD19 activation on a CD19/ICAM-1-functioned PDMS micropillar array. Force direction is defined by displacement vector projection relative to cell center; outward force is positive (spreading), inward is negative (contraction). Nonactivated CAR T cells derived from healthy donors cultured on micropillar arrays functionalized with ICAM-1 without CD19 were used as a nonactivated control. Data in d-g are collected from three healthy donors (n = 20 cells per donor), and data are normalized to T = 0 min. Thick lines denote mean, shaded areas denote s.e.m. (**h**) Oxygen consumption rate (OCR) of non-activated and activated CAR T cells measured using Seahorse Mito Stress Test with Oligomycin (Oligo, 0.5 μM), BAM15 (2.5 μM), and Rotenone + antimycin A (Rot/AA, 0.5 μM each) (n = 3 per group; 250,000 cells per well). (**i**) Energy phenotype profiling of CAR T cells reveals activation-associated shifts in metabolic preference (n = 3). (**j**) Correlation between total ATP production and mechanical energy expenditure ($E_{fe}$). (**k**) Correlation between glycolysis activation rate (calculated as the slope of ECAR change before and after oligomycin addition) and instantaneous force frequency. In J, K, each dot represents measurement from one healthy nonactivated donor (black) and three activated donors (red). In J, K, the black line is the linear regress fit. (**l**) Comparison of surface expressions of T cell activation marker (CD69, CD25) between activated (CD19/ICAM-1) and nonactivated (ICAM-1) CAR T cells (n=20 cells from 3 donors) and (**m**) ELISA quantification of cytotoxicity-related cytokines (IFNγ, TNFα, GZMB) 24 hours after CD19 activation. Data shown are from 9 or 10 technical replicates (n = 10 for IFNγ and TNFα; n = 9 for GZMB) from three healthy donors. CAR T cells seeded on substrate coated with only Poly-L-lysine are used as negative control (Blank). (**n**) Correlation between synaptic force and CD69 fluorescent intensity in CAR T cells. Each dot represented single cell measurements from Donor 1 and 2. Shaded circle represented distinct clusters in nonactivated and activated cell group. (**o**) Spearman’s correlation matrix shows correlations between the mechanical attributes ($f_{T}$ and $E_{\mathrm{free}}$), expression levels of CAR T cell activation markers (CD69 and CD25) and cytotoxicity-related cytokine markers (IFNγ and TNFα). Data is the mean value derived from 3 healthy donors. Legend bar shows Spearman’s coefficient, where a value closer to 1 indicates positive correlation and a value closer to −1 indicates negative correlations. Data in h-j are shown as the mean ± s.e.m. Statistical analysis in f and m used two-sided unpaired Student’s t-test, and in l used One-way ANOVA with Tukey’s post hoc test.

Mechanical forces are central to CAR T cell activation and cytotoxicity, requiring active cytoskeletal remodeling and force transmission at the immunological synapse (Fig. 1a)^37–39^. To quantify these dynamics, we exploited an elastic polydimethylsiloxane (PDMS) micropillar array substrate functionalized with CD19 antigen and intercellular adhesion molecule 1 (ICAM-1) (Extended Data Fig. 1a–b, **Supplementary Fig. 1**), mimicking the cancer cell surface while serving as mechanical sensors. Single anti-CD19 4–1BBζ-CAR T cells derived from healthy donors were imaged during antigen engagement, enabling simultaneous tracking of cell morphodynamics and synapse force based on pillar displacements (Fig. 1b–c, Extended Data Fig. 1c–d, **Supplementary Fig. 2**).

CAR T cells displayed a rapid biphasic “spreading–contraction” force response upon CD19 stimulation: an initial outward spreading phase over 3–4 minutes supported adhesion and synapse formation, followed by a stronger centripetal contractile force associated with CAR clustering and cytotoxic commitment (Fig. 1d–e). Morphodynamic analysis showed a concurrent increase in cell area during spreading, followed by contraction (Extended Data Fig. 1e–f). Compared to conventional TCR-activated CD8^+^ T cells, CAR T cells exerted greater synaptic forces at a faster rate (Extended Data Fig. 1c, f). In the absence of ICAM-1, synaptic forces were abolished (**Fig. S2**), confirming that LFA-1–ICAM-1 interactions are required for CAR T cell mechanical engagement.

To effectively capture the transient changes in subcellular dynamics across time and space, we applied a novel instantaneous frequency spectrum analysis, based on an adaptive decomposition of local instantaneous force dynamics of the measured single-cell force dynamics data (Extended Data Fig. 1g–h, details see Materials and Methods)^21, 40^. This analytic framework derives an instantaneous frequency *f*_*T*_ to characterize the cellular mechanoallostatic activity level, defined by the rate of cross bridge cycling within actomyosin complexes regulated by calcium activator ion to generate synaptic force upon activation. The spectrum analysis revealed that activated CAR T cells exhibited a significant shift to a higher instantaneous frequency *f*_*T*_ compared to the nonactivated group (Fig. 1f, **Supplementary Fig. 3**). This increase in *f*_*T*_ reflected the active engagement of cytoskeletal machinery in response to activation signal. Ultimately, the rate of cross bridge cycling within actomyosin complexes at the immunological synapse will impact on the efficiency of CAR centralization and cytolytic processes. Together, these findings identify a biphasic, high-frequency mechanoallostatic response as a hallmark of CAR T cell activation.

### CAR T cell activation is governed by mechano-energetic demand and supply

**Extended Data Fig. 2.**
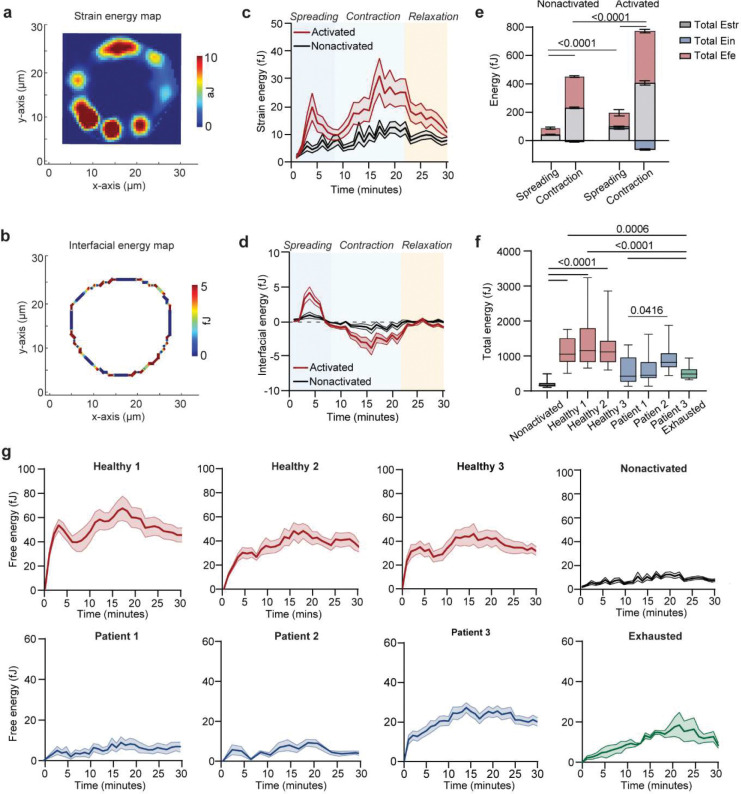
Profiling the energy landscape of CAR T cells upon activation. (**a**) Representative colorimetric maps of strain energy and (**b**) interfacial energy for a single CAR T cell on CD19-coated micropillar array. (**c**) Time-series evolution of normalized cellular strain energy ($E_{\text{str}}$), (**d**) interfacial energy ($E_{\text{int}}$) upon CAR T cell activation (n = 20 cells). Activated CAR T cells derived from healthy donors, patients, and the induced exhaustion group were seeded onto micropillar substrates functionalized with CD19 and ICAM-1. Nonactivated CAR T cells were seeded onto substrates coated with ICAM-1 only (**e**) Quantified area under the curve (AUC) of $E_{\text{str}}$ and $E_{\text{int}}$ during spreading and contraction phase of CAR T cells when encountering CD19-coated micropillars. Statistical analysis used two-sided unpaired Student’s t-test. (**f**) Box plot shows the comparison of total energy of CAR T cells consumed across individual donors. The box represents the interquartile range (IQR), with the median indicated by a line within the box. The whiskers extend to the minimum and maximum values. One-way ANOVA followed by Tukey’s post hoc test. (**g**) free energy of CAR T cells derived from individual patients (n = 20 cells). Data are shown as the mean ± s.e.m. All data was collected from three independent experiments.

We next investigated how the energetic demands of CAR T cell mechanical dynamics are met during activation. The biphasic “spreading–contraction” synaptic force pattern reflects distinct phases of cytoskeletal remodeling, each imposing specific energetic costs. To explore this, we quantify free energy ($E_{fe}$) of a cell, comprised of the cellular strain energy ($E_{\text{str}}$) expended to support synaptic force generation and an interfacial energy ($E_{\text{int}}$) expended to regulate the cellular shape as the cell adheres to and expands across a surface. The cellular strain energy $E_{\text{str}}$ is primarily associated with the synaptic force from cell-substrate adhesion^41^, which we approximate as: $E_{str}=\frac{1}{2}\sum_{i=1}^{N}{\vec{T}}_{i}(x,y)\cdot {\vec{u}}_{i}(x,y)$, where ${\vec{T}}_{i}(x,y)$ is the force at position (x,y), and ${\vec{u}}_{i}(x,y)$ is the displacement of micropillar at that position. Meanwhile, the interfacial energy ($E_{\text{int}}$) arising from the membrane tension and cortical forces at the cell periphery, drives cell morphological changes, balancing against cytoskeletal forces. As previously described^6^, the correlation between cell radius and cytoskeletal tension allows us to measure the average cellular interfacial tension (δ) in relation to the local cytoskeletal tension force (F) at the cell periphery, $\delta ∼\frac{\text{∮}\;Fdl}{L}$. Combining with the cell area (A) and roundness (r), we derive the equation for interfacial energy, assuming interfacial energy density is uniformly distributed at the cell periphery, as $E_{\mathrm{int}}=\delta *\sqrt{\frac{4\pi A}{r}}$ (Extended Fig. 2a–d). Together, these values describe the biophysical cost of mechanical work at the immunological synapse.

Upon CD19 stimulation, $E_{\text{str}}$ and $E_{\text{int}}$ rose sharply during the spreading phase, producing a peak in $E_{fe}$ that reflected the high energetic cost of adhesion and synapse formation (Fig. 1g). During contraction, $E_{\text{str}}$ increased further while $E_{\text{int}}$ declined, yielding a second energetic peak while stabilizing the cytolytic process by promoting a more compact synapse. Cells then relaxed toward a lower-energy baseline, consistent with resolution of the synapse. Following the fast-changing cycle of “spreading-contraction” phases, CAR T cells gradually return to an adaptive ground energy state, driven by the reduction both in strain and interfacial energies as they destabilize from the mature immunological synapse surface. The total free energy cost ($E_{tot}$) further revealed the high energy cost to activate CAR T cells (Extended Data Fig. 2e–f). These findings establish that CAR T cell mechanical allostasis is characterized by temporally resolved phases of energy demand, which might be facilitated by cellular metabolic processes of conversion of chemical to mechanical energy at the synapse.

**Extended Data Fig. 3.**
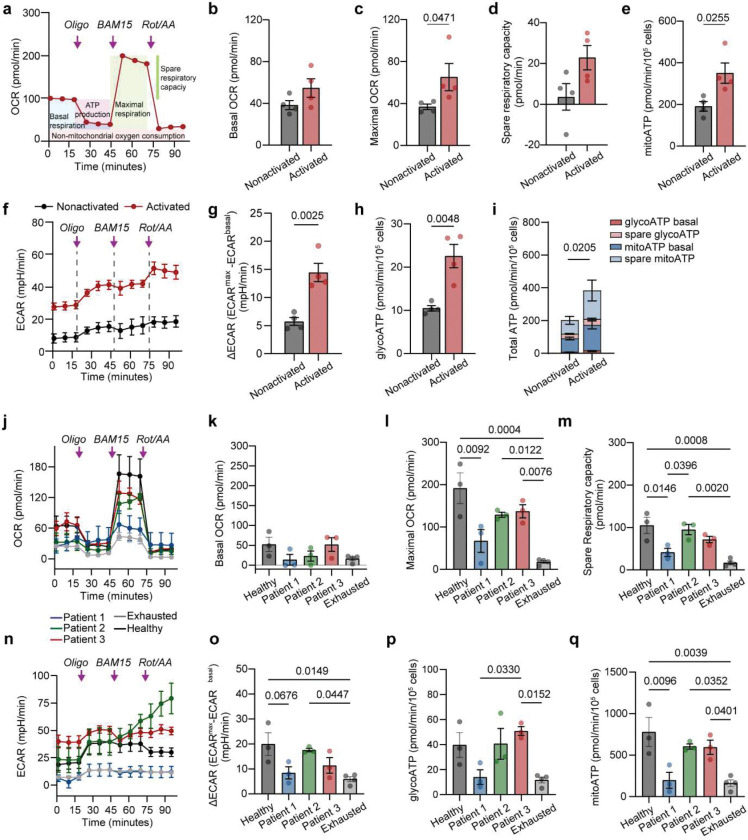
Measuring the metabolism of CAR T cells upon activation using Seahorse Mito Stress Test. **(a**) Representative OCR profile of CAR T cells measured using the Seahorse XF Mito Stress Test. Sequential injections of oligomycin (0.5 μM), BAM15 (2.5 μM), and rotenone/antimycin A (0.5 μM each) were used to probe mitochondrial respiration (n = 4 per group; 250,000 cells/well). (**b**) Quantification of basal OCR (average of first three time points), (**c**) maximal OCR (peak after BAM15), and (**d**) spare respiratory capacity (maximal minus basal OCR). (**e**) Mitochondrial ATP production rate derived from OCR after correcting for non-mitochondrial respiration. (**f**) ECAR profile of non-activated and activated CAR T cells under the same drug injection sequence (n = 4 per group, 250,000 cells/well). (**g-h**) ECAR increase post-oligomycin (ΔECAR), glycolytic ATP production are shown. (**i**) Component ATP production rate from glycolysis and oxidative phosphorylation (OXPHOS) under basal and spare conditions, calculated using a P/O ratio of 2.75. See Materials and Method for detailed ATP calculation. (**j**) OCR traces of healthy donor and patient-derived CAR T cells, including exhausted CAR T cells from chronic antigen stimulation. (**k–m**) Quantification of basal respiration, maximal respiration, and spare respiratory capacity across donor and patient CAR T cell groups. (**n**) ECAR traces across CAR T cell groups as in K. (**o**) ECAR increase postoligomycin (ΔECAR) as a measure of glycolytic capacity. (**p**) Mito and (**q**) GlycoATP production rates (glycolytic and mitochondrial) across groups. In j-p, data was collected from n = 5 for healthy, exhausted; n = 4 for patients, 250,000 cells/well. Data are shown as the mean ± s.e.m. P-values from two-tailed unpaired t-test or one-way ANOVA with multiple comparisons as indicated.

To examine how these demands are fueled, we profiled metabolic activity using Seahorse flux analysis. CD19 activation induced substantial increases in both oxygen consumption rate (OCR) and extracellular acidification rate (ECAR), consistent with coordinated upregulation of mitochondrial respiration and glycolysis (Fig. 1h, Extended Data Fig. 3a–g). Energy mapping revealed that activated CAR T cells transitioned into a highly energetic state, while nonactivated cells remained metabolically quiescent (Fig. 1i). Total ATP production was significantly increased in activated cells, suggesting that ATP output is upregulated to match the mechanical loads experienced during immunological synapse formation and activation (Fig. 1j, Extended Data Fig. 3e, h, i). Indeed, CAR T cells exhibiting high mechanoenergetic states, as defined by elevated free energy ($E_{fe}$), also demonstrated higher ATP production rates^42^, indicating tight coupling between bioenergetics and cytoskeletal demand. While mitochondria can produce greater quantities of ATP^42^, previous studies have emphasized the essential role of glycolysis during early activation and rapid effector responses^43^. We therefore hypothesized that the glycolysis changes (ΔECAR) may be critical for fueling early cytoskeletal dynamics and supporting rapid, serial target cell killing. Consistent with this, we observed that glycolytic changes strongly correlated with instantaneous force frequency in activated CAR T cells (Fig. 1k, Extended Data Fig. 3g), linking early metabolic reprogramming to the pace of actomyosin engagement at the immunological synapse. These findings underscore that mechanical adaptation in CAR T cells is not merely structural but bioenergetically demanding; wherein distinct metabolic pathways are coupled to force generation to meet the energetic loads of antigen engagement and cytotoxicity.

To establish the functional relevance of these mechano-metabolic signatures, we profiled CAR T cell effector functions, including expression of activation markers (CD69, CD25) and secretion of cytokines (IFN-γ, TNF-α, granzyme B) (Fig. 1l–m). Synaptic force generation positively correlated with CD25 expression (Fig. 1n). Moreover, mechanical energy and ATP production correlated with both activation (CD69, CD25) and cytokine release (TNF-α, IFN-γ) (Fig. 1o, **Supplementary Table 1**). These findings underscore that mechanical adaptation in CAR T cells is not merely structural but bioenergetically demanding; wherein distinct metabolic pathways are coupled to force generation to meet the energetic loads of antigen engagement and cytotoxicity.

### Mechano-metabolic signature is predictive of CAR T cell functional outcomes.

**Extended Data Fig. 4.**
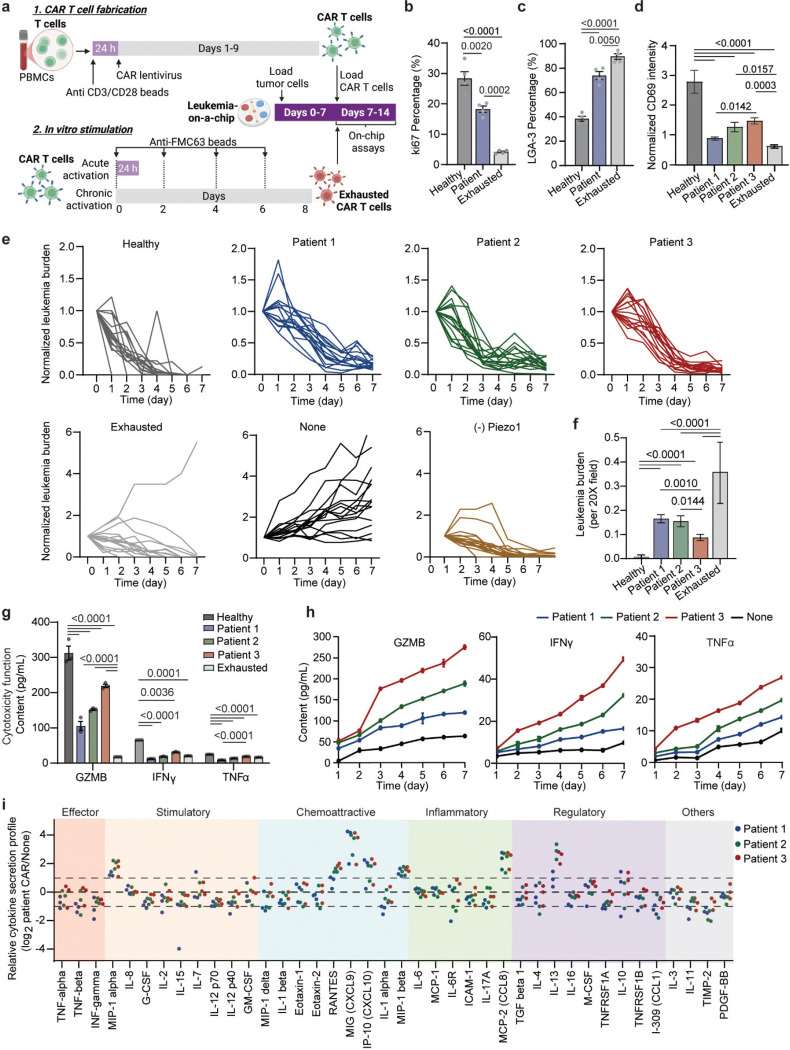
Functional characterizations of CAR T cells in a human leukemia-on-a-chip model. (**a**) Timeline of CAR T cell generation and on-chip assay workflow. T cells were activated with CD3/CD28 beads, transduced with lentivirus, and expanded prior to loading onto B-ALL-loaded chips for functional analysis. Schematic, Created in BioRender. (**b**) Flow cytometry quantification of percentage of cells expressing proliferation marker ki67 and (**c**) exhaustion marker LAG-3 across different groups of healthy donors, patient derived and exhausted CAR T cells (n = 4). (**d**) Quantification of CD69 expression in CAR T cells from healthy donors (n = 40 cells), patients 1–3 (n = 79, 81, 92 cells, respectively), and an exhausted CAR T group (n = 61 cells), based on pooled data from three technical replicates. (**e**) On-chip leukemia killing curves under treatment with patient, healthy donor derived, exhausted, and Piezo1-inhibited CAR T cells at low dose (1,250 cells per chip, effector to tumor cell ratio = 1:10). Each line represents leukemic cell count from a 20X field of view, normalized to cell count on D0. (**f**) Quantification of leukemia burden per 20X field under treatment of different donor-derived CAR T cells on Day 7. All bar graphs are presented as the mean and s.e.m. Data in e, f was collected from 4 technical replicates (n = 4) and 16 images per patient group. (**g**) Comparison of cytotoxicity-related cytokines (IFNγ, TNFα, GZMB) across different groups of patients, exhausted, and healthy donor derived CAR T cells after on-chip interaction for 2 days (n = 3). (**h**) ELISA measured IFN-γ, TNF-α, GMZB secretions from leukemia chips treated with patient CAR T cells at low dose (1250 T cells per chip) or without CAR T cells (None) at different time points (n = 3). (**i**) Comparison of cytokine secretion profiles on-chip 2 days after treatment with different patient derived CAR T cells at high dose (10,000 CAR T cells per chip). Cytokine profiles were measured by a Human Inflammation Array C3 membrane kit and were normalized to chips without CAR T cell treatment. Statistical analysis in b, c, d, f and g used One-way ANOVA with Tukey’s post hoc test.

**Figure 2.**
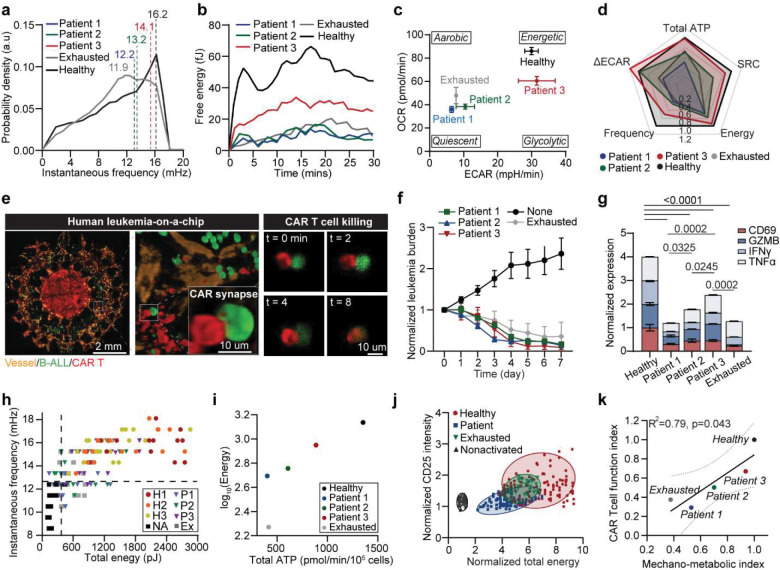
Mechanoenergetic signature is predictive of CAR T cell functional outcomes. (**a**) Instantaneous frequency distributions of force dynamic responses, (**b**) change in total free energy of CAR T cells from 3 patient donors, induced exhaustion, and one healthy donor (donor 1) from 0 to 30 min upon CD19 activation (n = 20 cells for each donor). Data in A-C were normalized to values at T = 0 min. Thick lines represent the mean per donor from three independent experiments, corresponding to **Extended Data Fig. S2–3**. (**c**) Energy phenotype profiling from glycolysis and oxidative phosphorylation (OXPHOS) across CAR T cell groups measured from Seahorse Mito Stress Test (n = 3). (**d**) Comprehensive profiling of mechano-metabolic reprogramming in CAR T cells, comprising mechanical parameters (energy and instantaneous frequency) and metabolic parameters (total ATP production from OXPHO and glycolysis, glycolysis activation rate, and spare respiratory capacity). All data was normalized to respected average value in healthy group. (**e**) Left panel: Representative immunofluorescence images showing CAR T cell (red, Vybrant DiD) and B-ALL cell (green, GFP) in a vascularized (yellow, CD31) human leukemia-on-a-chip. The insert shows formation of immunological synapse between a CAR T cell (red) and a B-ALL cell (green). Right panel: Representative images showing the process of a CAR T killing a CD19^+^ B-ALL cell. (**f**) On-chip response curves and (**g**) quantification of leukemia burden per 20X field and functional response (activation, cytokine secretion) under treatment of patient-derived CAR T cells at a dose of 1,250 (effector to tumor cell ratio = 1:10). Statistical analysis used One-way ANOVA with Tukey’s post hoc test. (**h**) Correlation between the instantaneous frequency and total energy ($E_{fe}$) of CAR T cells (n = 20 for each donor) derived from healthy donors (H1, H2, H3), patient (P1, P2, P3) donors, and exhausted group (Ex). Total energy is derived from quantified areas under the curve (AUC) of free energy response curve for the first 30 min upon CAR T cell activation with the CD19/ICAM-1-coated micropillars. Nonactivated CAR T cells derived from healthy donors cultured on micropillar arrays functionalized with ICAM-1 without CD19 were used as a nonactivated control (NA). (**i**) Correlation between total energy and ATP production rate among CAR T cell groups. (**j**) Correlation between the surface expression level of T cell activation marker CD25 and the free energy derived from cell area and synaptic force of single CAR T cells 24 hours after seeded on micropillar arrays. Each data point represents a single cell; shaded regions indicate group clusters. (**k**) Correlation between CAR T cell mechano-metabolic index and function index. The composite mechano-metabolic index was derived by integrating multiple parameters that characterize CAR T cell mechano-metabolic function: energy ($E_{fe}$), instantaneous frequency ($f_{T}$), total ATP production ($M_{\mathrm{ATP}}$), glycolysis activation rate ($M_{\mathrm{glyco}}$), spare respiratory capacity ($M_{\mathrm{mito}}$). The CAR T cell function index was derived by integrating cytotoxic efficiency ($C_{L}$), level of activation ($A_{\mathrm{CD69}}$), and quantification of activation associated GZMB, IFNγ, TNFα cytokine secretion profile ($S_{\mathrm{Cyt}}$) from leukemia chip treated with CAR T cells. The indices were normalized to a healthy control (healthy donor 1). Each normalized index was expressed as a percentage of its reference value. Normalized indices were then categorized into performance tiers based on fixed thresholds (see Materials and Methods). Each dot represents a group. Solid line shows linear regression; shaded area shows 95% confidence interval. Statistical comparisons in g were performed using one-way ANOVA with Tukey’s post hoc test.

We next comparatively study the mechano-metabolic signatures of different patient-derived CAR T cells, along with exhausted CAR T cells that have undergone serial expansions with activation and re-stimulation with anti-FMC63/ICAM-1-coated beads (Extended Data Fig. 4a, details see Materials and Methods).^44^ Succesful induced exhaustion was confirmed by elevated expression level of exhaustion marker LAG-3 and decreased secretion of effector cytokines (GZMB, IFNγ, TNFα) and memory-associated cytokines (IL2, IL15), along with a decrease in proliferation marker ki67 (Extended Data Fig. 4b–c, **Supplementary Fig. 4**). Compared to healthy donor-derived cells, patient-derived CAR T cells exhibited heterogeneous and attenuated mechanical dynamics, with reduced force magnitude and frequency (Fig. 2a–b). Energy mapping confirmed distinct metabolic states: healthy CAR T cells showed robust OCR and ECAR, while patients 1–2 were metabolically quiescent and patient 3 retained partial metabolic capacity (Fig. 2c–d, Extended Data Fig. 3j–p). Exhausted CAR T cells displayed uniformly diminished ATP output. Composite radar plots integrating ATP, spare respiratory capacity, energy, force frequency, and glycolytic activity distinguished high-performing (healthy, patient 3) from dysfunctional (patients 1–2, exhausted) profiles (Fig. 2d).

Furthermore, we evaluated the functional outcomes of these CAR T cell products using a 3D microfluidic-based human ‘Leukemia-on-a-Chip’ *in vitro* tumor model (details see Materials and Methods). This previously developed ‘Leukemia-on-a-Chip’ model recapitulated *in vivo* leukemic bone marrow niche, which allowed for a preclinical evaluation of chemotherapy^45^ and CD19 CAR T cell therapy^46, 47^ (Fig. 2e). After 7 days of on-chip treatment, healthy and patient CAR T cells-controlled leukemia progression, while exhausted cells showed diminished killing (Fig. 2e–f, Extended Data 4d–f). Cytokine secretion mirrored these outcomes (Fig. 2g, Extended Data 4g–i). Clustering analysis of free energy versus force frequency identified an energetic barrier that separated functional from dysfunctional cells (Fig. 2h–i). Activated CD25 expression similarly clustered with high-energy states (Fig. 2j). To unify mechanical and metabolic traits, we derived a composite mechano-metabolic index (MI) incorporating five parameters (details see Materials and Methods). The MI correlated strongly with the CAR T cell function index (FI) based on cytotoxicity, cytokine secretion, and activation marker expression (R^2^ = 0.79, p = 0.043) (Fig. 2k), demonstrating that the mechano-metabolic signature predicts CAR T cell efficacy.

### Piezo1 integrates mechanical and metabolic reprogramming through a Ca^2+^-Wnt–Rac1 signaling axis.

**Extended Data Fig. 5.**
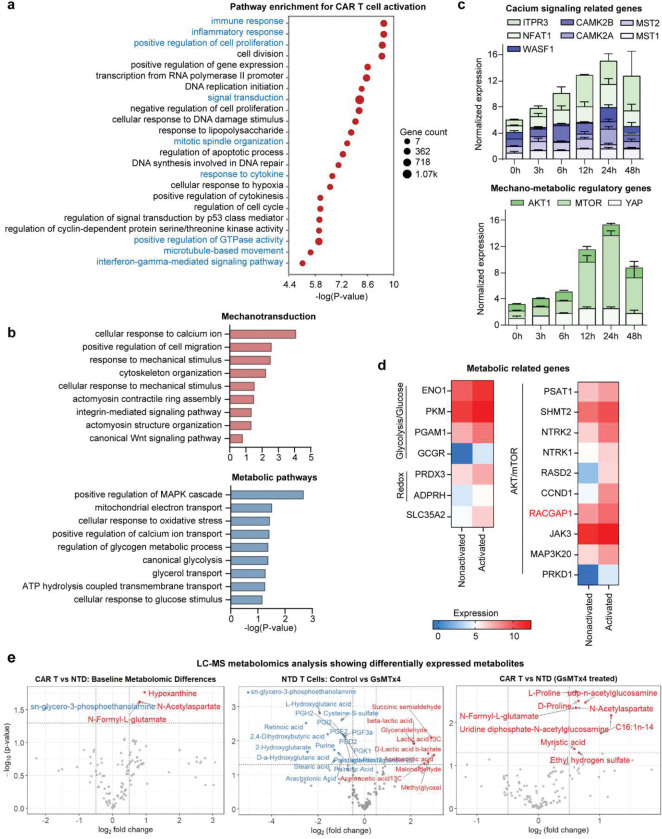
Transcriptomic and metabolomic analyses reveal mechano-metabolic associated pathways in CAR T cell activation. (**a**) Total RNA-seq Gene ontology (GO) enrichment analysis showing the top enriched GO biological process terms in activated CAR T cells. Activated CAR T cells cultured on CD19/ICAM-1-coated substrates compared to blank (Poly-L-lysine coated) controls. Highlighted pathways are directly related to CAR T cells immune and mechano-responses. (**b**) Total RNA-seq Pathway enrichment analysis highlighting mechanotransduction and metabolic programs in CAR T cells. Data was collected from 2 technical replicates per condition. (**c**) RT-qPCR quantification of Piezo1/Ca^2+^ signaling related gene expression (top) and mechano-metabolic (PI3K–AKT and Hippo pathways) regulatory genes (bottom) upon CAR T activation. (**d**) Total RNA-seq analysis heatmap showing total expression level of genes involved in CAR T metabolic pathway. (**e**) Volcano plot from LC-MS metabolomics analysis showing differentially expressed metabolites in non-transduced (NTD) T cells and CAR T cells with or without GsMTx4 treatment in LC-MS (n = 4 for NTD, n = 3 for NTD-GsMTx4, CAR, CAR-GsMTx4). Log2 fold change < 0 means a metabolite is downregulated in the inhibitor-treated groups. Cutoff for significance is chosen at |log2FC|>0.5 and p-value <0.05. Points in red are upregulated, while points in blue are downregulated metabolites of significance according to the criteria above.

**Extended Data Fig. 6.**
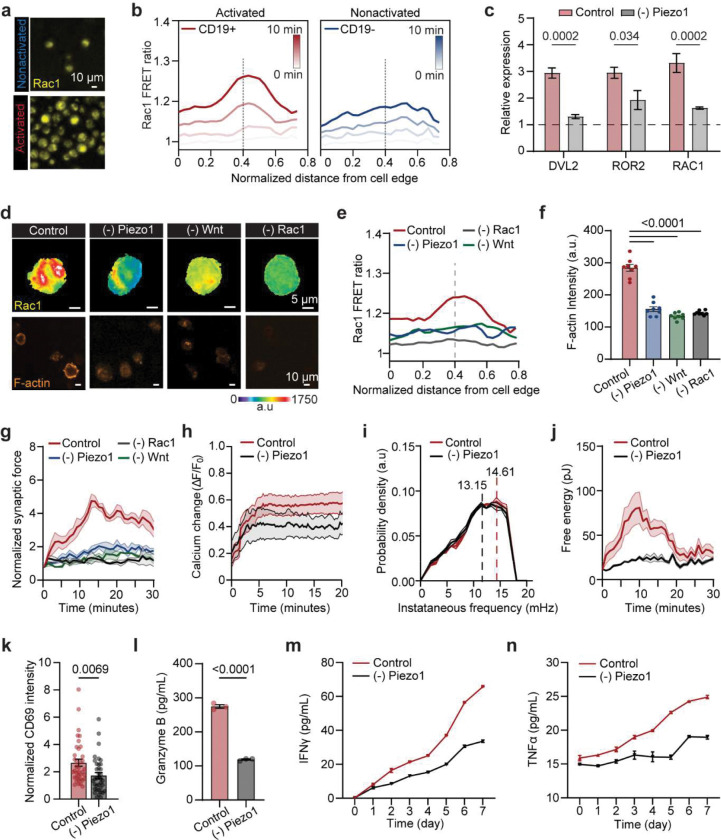
Piezo1 regulates CAR T cell cytoskeletal and mechano-energetic dynamics. (**a**) Representative radiometric FRET-Rac1 images and (**b**) spatial distribution of Rac1 in nonactivated and activated CAR T cells within 10 minutes contact with ICAM-1-coated (CD19−) and CD19/ICAM-1-coated (CD19+) coverslip. (**c**) RT-qPCR results showing changes of Wnt pathway regulators in CAR T cells after treatment with GsMTx4 for 30 minutes (Piezo1(−)) comparing to healthy CAR T cells (control). (**d**) Representative images of Rac1 FRET ratio and F-actin (Phalloidin), (**e**) representative radiometric quantification showing Rac1 spatial distribution in pharmacologically treated CAR T cells (2.5 μM GsMTx4 for 30 mins Piezo1 inhibition, 25 μM IWP2 for 24 hours for Wnt inhibition, 100 μM NSC23766 for 24 hours Rac1 inhibition) and in healthy CAR T cells (control). Rac1 images were captured within 30 minutes upon CAR T cells contact with CD19/ICAM-coated coverslip, while F-actin (Phalloidin) was fixed and fluorescent stained 3 hours after encountering CD19/ICAM-coated coverslip. (**f**) Quantification of F-actin expression (n = 5 technical replicates) and (**g**) synaptic force dynamics of pharmacologically treated CAR T cells (n = 8 cells, 2 independent experiments). Data in g was normalized to the baseline force at T = 0 min. (**h**) Time course measurement of intracellular calcium measured by Fluo4 AM intensity in healthy control and GsMTx4-treated CAR T cells upon encountering CD19/ICAM-1-coated coverslip (n = 15 cells). (**i**) Instantaneous force frequency and (**j**) free energy measured in control healthy and pharmacologically treated CAR T cells (n = 8 cells). Data was collected from two independent experiments. (**k**) Fluorescent quantification of CAR T cell activation markers CD69 (n = 37 cells from 3 technical replicates), (**l**) ELISA measured TNFα, (**m**) INF-γ, and (**n**) Granzyme B from leukemia chips (n = 3 technical replicates). All data are shown as the mean ± s.e.m. Statistical analysis in c, k and n used two-sided unpaired Student’s t-test, and in f used One-way ANOVA with Tukey’s post hoc test.

**Figure 3.**
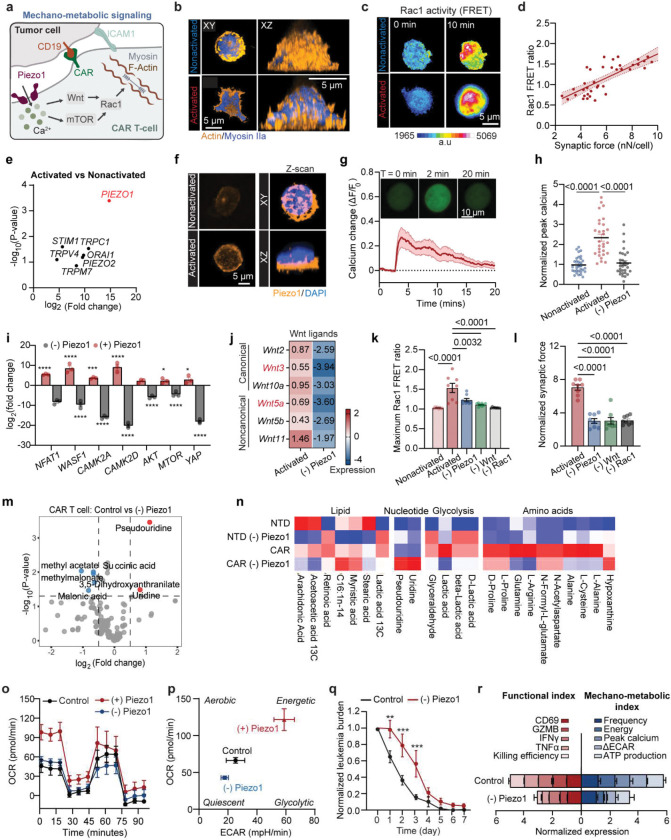
Piezo1/Wnt/Rac1 mechano-signaling pathway regulates CAR T cell mechano-metabolism and function. (**a**) Schematic showing mechano-metabolic signaling pathway in CAR T cell activation. (**b**) Representative confocal immunostaining images showing the spatial distributions of actin and myosin, and (**c**) FRET images showing spatial distribution of Rac1 in activated CAR T cells upon CD19 activation on a CD19/ICAM-1-functioned glass substrate and nonactivated cultured on substrate functionalized with ICAM-1 without CD19. (**d**) Linear correlation between Rac1 activity and synaptic force. Linear regression fit shown with 90% CI (n = 58 cells, from 3 independent experiments). (**e**) RT-qPCR analysis showing differential expression (log2) and p value of mechanosensitive channel transcripts between activated and nonactivated CAR T cells. Cells were collected after 1-hour contact with the CD19/ICAM-1 and ICAM-1 functioned glass substrates. (**f**) Representative confocal immunostaining images showing the spatial distributions of Piezo1 in activated and nonactivated CAR T cells 30 min after in contact with the CD19/ICAM-1 and ICAM-1 functioned glass substrates. (**g**) Time course measurement of Ca^2+^ change as CAR T cells contact with the ICAM-1/CD19-coated glass substrates (n = 30 cells). Data was normalized to T = 0 min. The thick line represents the mean and the shaded area represents s.e.m. (**h**) Peak calcium response as activated and nonactivated CAR T cells cultured on CD19/ICAM-1 and ICAM-1 functioned substrates (n = 30 cells). (−) Piezo1 group is CAR T cells cultured on CD19/ICAM-1 functionalized substrates and pre-treated with 2.5μM GsTMx4 for 30 mins. (**i**) RT-qPCR quantification showing relative expression of mechano-metabolic regulator in response to Piezo1 inhibition with GsMTx4 ((−) Piezo1) and Piezo1 enhancement by pre-treatment with 0.3 μM Yoda1 for 30 mins ((+) Piezo1) (n = 3 technical replicates). (**j**) RT-qPCR quantification showing relative expression of Wnt signaling regulators in CAR T cells under inhibition of Piezo1 by pre-treatment with 2.5μM GsTMx4 for 30 mins (n = 3 technical replicates). (**k**) Quantification of maximum Rac1 FRET signal across nonactivated, activated, and pharmacologically treated CAR T cells (2.5 μM GsMTx4 for 30 mins Piezo1 inhibition, 25 μM IWP2 for 24 hours for Wnt inhibition, 100 μM NSC23766 for 24 hours Rac1 inhibition; n = 8 cells, 3 independent experiments). (**l**) Synaptic force dynamics of pharmacologically treated CAR T cells (n = 8 cells, 2 independent experiments). Data in was normalized to the baseline force at T = 0 min. (**m**) Untargeted LC-MS metabolomics analysis of non-transduced T cells (NTD) and CAR T cells with and without treatment with GsMTx4. Log2 fold change < 0 means a metabolite is downregulated in the inhibitor-treated groups MS (n = 4 for NTD, n = 3 for NTD (−) Piezo1, CAR, CAR (−) Piezo1). Cutoff for significance is chosen at |log2FC|>0.5 and p-value <0.05. Points in red are upregulated, while points in blue are downregulated metabolites of significance according to the criteria above. (**n**)Heatmaps of differentially abundant metabolites between among groups. Data are normalized within each row (compound) using z-score normalization. (**o**) OCR trace and (**p**) energy phenotypic profile shows metabolic changes in CAR T cells upon inhibition of Piezo1 by GsTMx4 ((−) Piezo1, n = 4). (**q**) On-chip response curves of leukemia burden per 20X field under treatment of CAR T cells or GsMTx-4 treated CAR T cells ((−) Piezo1) at a dose of 1,250 cell per chip (n = 4 technical replicates). (**r**) Composite mechanical and functional indices of CAR T cells in control and Piezo1-inhibited group ((−) Piezo1). The mechanical index comprises instantaneous force frequency (n = 8 cells), free energy (n = 8 cells), and peak calcium change (n = 20 cells). The CAR T cell function index comprises surface activation markers CD69 after on-chip interaction with leukemia blasts for 2 days (n = 37 from 3 technical replicates), ELISA measured secretions of TNF-α, INF-γ, and Granzyme B from leukemia chips (n = 3 technical replicates). All data was normalized to the average measured values of the control healthy group. All data are shown as mean ± s.e.m. Statistical tests in h, i, k, l used one-way ANOVA with Tukey’s post hoc test, and in q used unpaired two-tailed Student’s t-test. * denotes p<0.05, ** denotes p<0.005, *** denotes p<0.0005, **** denotes p<0.00005.

To define how mechanical inputs interface with metabolic programming in CAR T cells, we profiled transcriptional and cytoskeletal changes during antigen stimulation. Total RNA sequencing (RNA-Seq) revealed strong enrichment of immune activation programs, including cytokine secretion, inflammatory responses, alongside mechanotransduction pathways such as calcium signaling and cytoskeletal remodeling (Fig. 3a, Extended Data Fig. 5a–d, **Supplementary Fig. 5**). Actin imaging confirmed extensive concentration at the synapse in activated versus resting cells (Fig. 3b). Consistent with this, RACGAP1—a Rac1 modulator^48^ critical for actin cytoskeleton remodeling, was among the most upregulated genes (Extended Data Fig 5d)^49^. To observe Rac1 dynamics, we utilized FRET-based Rac1 biosensor^50^ and found robust Rac1 activation localized to the immunological synapse, scaling with force generation over time (Fig. 3c–d, Extended Data Fig 6a–b). Furthermore, activated cells shifted to a more energetic phenotype, with upregulation of glycolytic enzymes (ENO1, PKM, PGAM1), redox regulators (PRDX3, ADPRH), and AKT–mTOR pathway components (PSAT1, SHMT2, JAK3, RACGAP1) (Extended Data Fig. 5d) ^51^. Pathway analysis highlighted mitochondrial respiration, glycolysis, and calcium ion transport as major nodes of enrichment (Extended Data Fig. 5b).

Screening of calcium-regulated ion channels identified Piezo1 as the most strongly upregulated upon CAR activation across all donors (Fig. 3e). Immunostaining confirmed its localization at the immunological synapse and association with elevated intracellular Ca^2+^ (Fig. 3f–h). Meanwhile, pharmacological inhibition with GsMTx4 reduced antigen-triggered Ca^2+^ flux (Fig. 3h). Perturbation of Piezo1 signaling selectively altered the expression of downstream effectors: calcium regulators (NFAT1, WASF1, and CAMK2A) and mechano-metabolic regulators (AKT, MTOR, YAP) were downregulated upon inhibition, whereas activation induced their upregulation (Fig. 3i)^52^. In CD8^+^ T cells, we also observed upregulation of Piezo1 expression upon TCR activation (**Supplementary Fig. 6a-c**). Pharmacological activation by Yoda1 showed that Piezo1-mediated Ca^2+^ influx was stronger in CAR compared to CD8^+^ T cells (**Supplementary Fig. 6d**).

Piezo1 further engaged Wnt signaling, where both canonical ligands (e.g., Wnt3a) and non-canonical ligands (e.g., Wnt5a) were induced during CAR activation and suppressed by Piezo1 blockade (Fig. 3j). Inhibition of Piezo1 abrogated downstream transcriptional programs of both β-catenin–dependent and Ca^2+^-dependent Wnt pathways (Extended Data Fig. 6c). Functionally, suppression of Piezo1, Wnt, or Rac1 reduced Rac1 activity, diminished F-actin intensity, and impaired synaptic force generation (Fig. 3k–l, Extended Data Fig. 6d–g). This decline in force response in Piezo1-inhibited cells was also observed in CD8^+^ T cells (Extended Data Fig. 6e–f). Together, these results position Piezo1 as an upstream mechanosensor that couples Ca^2+^ influx to Wnt–Rac1 signaling, thereby coordinating cytoskeletal remodeling and metabolic reprogramming required for CAR T cytotoxicity.

### Blocking of Piezo1 rewired intracellular metabolism.

To define how Piezo1 shapes fuel utilization, we profiled intracellular metabolites by liquid chromatography mass spectrometry (LC-MS) metabolomics analysis. At first sight, CAR T cells displayed higher biosynthetic demand with upregulated levels of N-acetylaspartate, N-formyl-L-glutamate, and hypoxanthine, compared to non-transduced T cells, suggesting higher nucleotide salvage and amino acid metabolism to support proliferation and killing (Extended Data Fig 5e). Upon Piezo1 inhibition in conventional T cells, we observed suppression of lipid-derived mediators and oxidative intermediates together with a marked rise in lactate (D-, β- and total), glyceraldehyde, acetoacetate, methylglyoxal, and malondialdehyde, indicating a shift toward fermentative metabolism accompanied by stress-associated carbonyl by-products (Extended Data Fig 5e). In striking contrast, the omics data suggested that Piezo1 activity licenses mitochondrial TCA/OXPHOS and aspartate supply, while its blockage corresponds to adenine nucleotide breakdown and energy stress in CAR T cells. In particular, we found coordinated depletion of TCA intermediates (e.g., succinate, fumarate, citrate), reductions in multiple amino acids (arginine, alanine, cysteine, tryptophan) and hexose phosphates, while the pyrimidine nucleosides uridine and pseudouridine increased (Fig. 3m–n). These shifts were accompanied by reductions in oxygen consumption, glycolysis, and ATP production measured in Seahorse assay (Fig. 3o–p, r), Interestingly, Piezo1 inhibition seems to further unmask metabolic programs that distinguish CAR T cells, possibly due to their engineered state or higher metabolic demand.

Functionally, Piezo1 blockade compromised calcium signaling, synaptic force frequency, and cytotoxic capacity, resulting in impaired tumor clearance in an on-chip leukemia model (Fig. 3q, Extended Data Fig. 6i–o). These cells also showed reduced activation and effector cytokine secretion, a phenomenon also observed in CD8^+^ T cells (Fig. 3r, **Supplementary Fig. 6a-i**). Together, our data argues that Piezo1 serves as a metabolic checkpoint that couples mechanotransduction to fuel selection and its inhibition compromises CAR T metabolic fitness and effector function. CRISPR gene editing further showed that Piezo1 Gain-of-function increased activation of T cells in vitro (**Supplementary Fig. 6J-M**). Furthermore, Piezo1 activation with Yoda1 increased basal respiration but unexpectedly abolished spare respiratory capacity, indicating that mitochondria operated near maximal rates at baseline. Thus, both inhibition and hyperactivation of Piezo1 disrupt cellular bioenergetics—through insufficient fuel input in one case and mitochondrial overdrive in the other—pointing to the existence of an optimal, intermediate range of Piezo1 activity required to sustain CAR T cell fitness.

### Piezo1 signaling follows a non-linear “Goldilocks” model that defines CAR T cell function.

**Figure 4.**
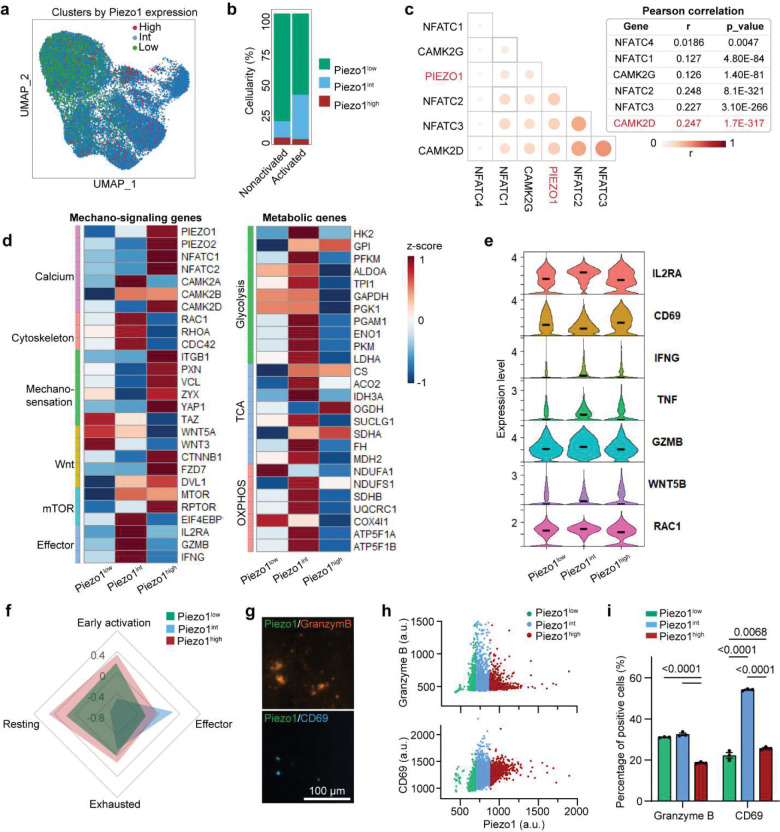
Intermediate level of Piezo1 is optimal for CAR T cell mechano-metabolic and effector functions. (**a**) UMAP projection of scRNA-seq data showing clustering of CAR T cells based on Piezo1 expression levels, categorized into Low, Intermediate, and High groups (Piezo1^low^, Piezo1^int^, Piezo1^high^) by k-means clustering (k = 3). (**b**) Bar plot showing the proportion of Piezo1^low^, Piezo1^int^, Piezo1^high^ expression groups within activated and non-activated CAR T cell populations. (**c**) Correlation of Piezo1 with downstream calcium–NFAT signaling genes. Pearson correlation matrix of Piezo1 expression with NFAT family transcription factors (NFATC1–4) and CaMKII isoforms (CAMK2D, CAMK2G). (**d**) Heatmap showing z-score normalized expression of selected genes related to mechanosignaling (left) and metabolic pathways (right) across Piezo1 expression groups. Piezo1^int^ group is associated with increased expression of effector-associated genes, cytoskeletal regulators, mTOR signaling components, glycolytic genes (e.g., HK2, ALDOA), and OXPHOS genes (e.g., COX4I1, NDUFS1). (**e**) Violin plots showing single-cell expression of selected effector and signaling genes across Piezo1 expression groups. Piezo1^high^ cells show elevated expression of IL2RA, CD69, IFNG, TNF, and GZMB. (**f**) Radar plot depicting the distribution of T cell functional states (resting, early activation, effector, exhausted) across Piezo1 expression groups. (**g**) Representative staining images of Piezo1, CD69 and Granzyme B in single CAR T cells, and (**h**) Scatter plot of Granzyme B expression versus Piezo1 levels (top), and CD69 expression versus Piezo1 levels (bottom) measured by a microfluidic digital ELISA single-cell phenotyping platform. Cells are divided into 10 domains according to their Piezo1 level using K-mean clustering. Domains 1–2 are denoted as Piezo1^low^, domains 3–5 are denoted as Piezo1^int^, and domain 5–10 are denoted as Piezo1^high^ (n = 707, 2326, 1663 cells for Low, Intermediate, and High Piezo1 clusters respectively). (**i**) Percentage of positive cells expressing Granzyme B and CD69 in the clustered cells. A threshold was determined according to the data from negative control. Cells with signals larger than the threshold are considered positive (n = 3). Data are shown as the mean ± s.e.m. Statistical tests used one-way ANOVA with Tukey’s post hoc test.

To explore how Piezo1 expression relates to CAR T cell performance, we stratified cells into low, intermediate, and high Piezo1-expressing states using single-cell RNA sequencing (scRNA-seq) analysis (Fig. 4a). Healthy activated CAR T cells were enriched for intermediate Piezo1 states, whereas low Piezo1 expression dominated the non-activated pool (Fig. 4b). Correlation analysis revealed that Piezo1 expression was significantly associated with calcium-dependent transcriptional programs, including NFAT family members and CaMKII isoforms, with strongest correlation observed for CAMK2D and NFATC3 (Fig. 4c). These associations place Piezo1 upstream of a Ca^2+^–CaMKII–NFAT axis, consistent with its role as a mechanosensitive ion channel.

We then mapped mechano-signaling and metabolic pathway output across Piezo1 states. High Piezo1 cells upregulated cytoskeletal regulators (RAC1, RHOA, CDC42), calcium effectors (CAMK2A/B/D, NFATC2), and Wnt/mTOR components, while also increasing glycolytic (HK2, PKM, LDHA) and OXPHOS genes (COX4I1, NDUFS1) (Fig. 4d). Yet, the most coherent and balanced induction occurred at intermediate Piezo1: these cells showed coordinated activation and effector programs (IL2RA, CD69, IFNG, TNF, GZMB) together with mechano-regulators (WNT5B, RAC1) (Fig. 4e). State mapping aligned the intermediate group with early activation and effector fates, whereas low Piezo1 remained resting and high Piezo1 shifted toward exhaustion (Fig. 4f). We hypothesize a non-linear “Goldilocks” setpoint in which intermediate Piezo1 optimizes CAR T cells function by (1) producing transient Ca^2+^ flux of sufficient magnitude to engage CaMKII–NFAT without triggering Ca^2+^ overload, (2) calibrates actin dynamics and organization to avoid over-softening or stiffening of the cytoskeleton, and (3) maintain a balance ratio between glycolysis and OXPHOS to meet biosynthetic demand without tipping into energy stress. We then employed a microfluidic digital ELISA platform, adapted from the previously reported PEdELISA system^53–55^ to analyze single cell Granzyme B secretion and correlate it with Piezo1 expression (Fig. 4g–i, details see Materials and Methods). At the protein level, intermediate Piezo1 expression correlated with maximal Granzyme B production and activation, while high Piezo1 cells displayed diminished cytokine expression and activation, consistent with signaling saturation and metabolic stress (Fig. 4g–i). Together, these data establish that Piezo1 is necessary but not sufficient for optimal CAR T cell function. Instead, an intermediate “Goldilocks zone” of Piezo1 signaling emerges as the optimal state for sustaining CAR T cell activation, mechano-metabolism, and cytotoxicity.

### Both hypoactive and hyperactive Piezo1 states impair mechano-metabolic fitness and drive dysfunction in patient and exhausted CAR T cells.

**Extended Data Fig. 7.**
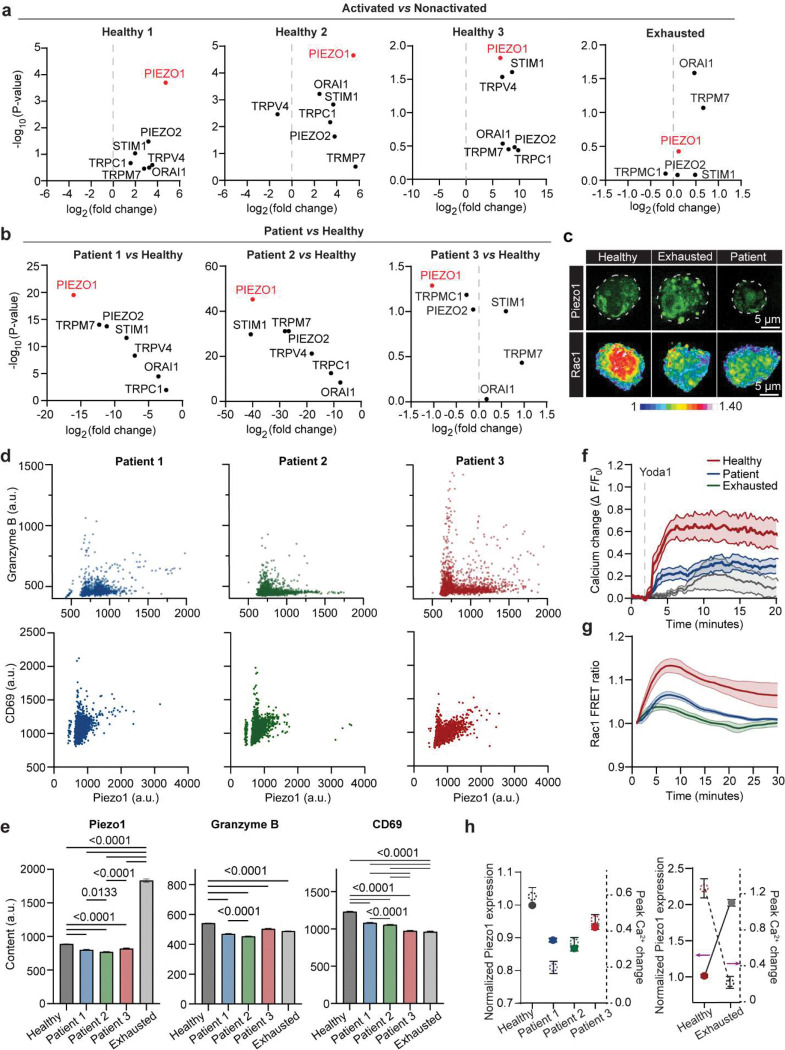
Aberrant Piezo1-medaited mechano-metabolic signaling in patient and exhausted CAR T cells. (**a**) RT-qPCR analysis showing differential expression (log2) and p value of mechanosensitive channel transcripts in CAR T cells derived from activated and nonactivated CAR T cells derived from individual healthy donors and exhaustion-induced cells, and (**b**) in patient-derived versus healthy CAR T cells. Each dot represents average values of gene expression from 3 technical repeats (n = 3). (**c**) Representative images of Piezo1 staining and Rac1 spatiotemporal distribution in healthy, patient, and exhausted CAR T cells. (**d**) Microfluidic digital ELISA analysis of single-cell correlation between Granzyme B intensity and Piezo1 (top) and between CD69 intensity and Piezo1 (bottom), and (**e**) quantification of single-cell Piezo1, Granzyme B and CD69 levels across CAR T cells groups. In d and e, n = 4696, 2403, 3489, 2657, 1021 cells for healthy, patient 1, patient 2, patient 3, exhausted group respectively. One-way ANOVA followed by Turkey Post Hoc test. (**f**) Comparison of time course Ca^2+^ influx in response to Yoda1 stimulation measured by Fluo4 AM intensity (n = 20 cells), and (**g**) the Rac1 activity dynamics after CD19 activation (n = 10 cells) across healthy, patient-derived, and exhausted CAR T cells. The thick line represents the mean and the shaded area represents s.e.m. (**h**) Comparison of Piezo1 expression and peak Piezo1-mediated Ca^2+^ response across healthy, patient-derived, and exhausted CAR T cells. Data were presented as mean and s.e.m. and normalized to the average values of healthy CAR T cells.

**Figure 5.**
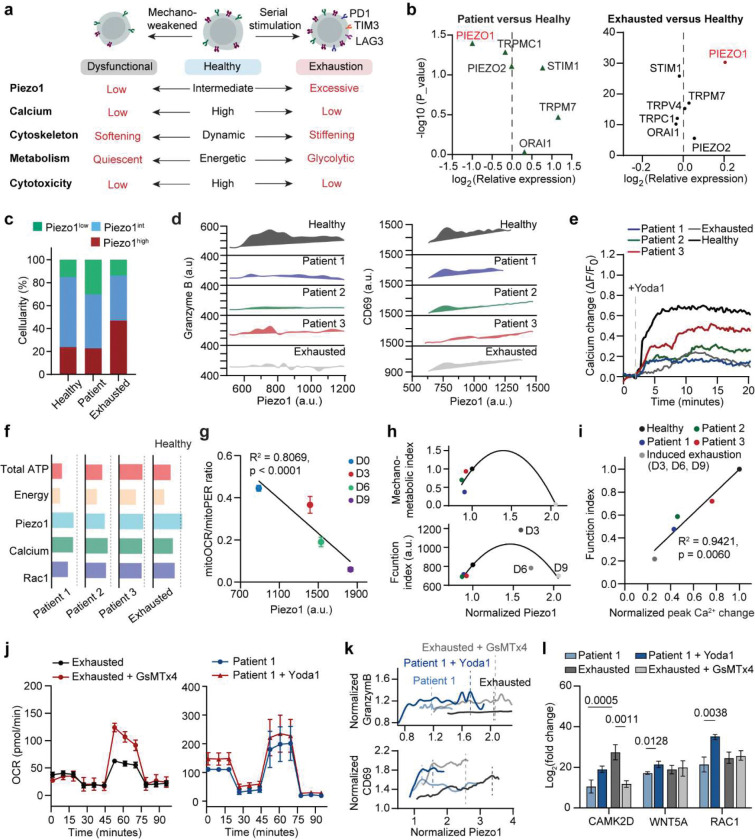
Aberrant Piezo1-mediated mechano-metabolic signaling in dysfunctional and exhausted CAR T cells. (**a**) Schematic representation of deviations of Piezo1-dependent mechano-metabolic signaling in dysfunctional and exhausted CAR T cells. (**b**) RT-qPCR analysis showing differential expression (log2) and p value of mechanosensitive channel transcripts in CAR T cells from patient-derived and induced exhaustion versus healthy CAR T cells. Data is summarized from three patients. (**c**) Cellularity plot showing abundance of low, intermediate, and high Piezo1-expressing cells among all groups measured by microfluidic digital ELISA single-cell phenotyping platform. Piezo1 expression in each group was stratified into Low, Intermediate, and High categories using the K-mean clustering threshold defined in Fig. 4h. (**d**) Correlation of Granzyme B expression versus Piezo1 level (left) and CD69 expression versus Piezo1 level (right) across healthy, patients, and exhausted CAR T cells during a 9-day serial stimulation, measured by microfluidic digital ELISA single-cell phenotyping platform. (**e**) Average time-course intracellular calcium response measured by Fluo4 AM intensity in healthy, individual patient-derived, and exhausted CAR T cells in response to Yoda1 stimulation (n = 20 cells). (**f**) Bar chart shows mechano-metabolic profiles (total ATP, free energy, Piezo1 expression, peak calcium change, and peak Rac1) of CAR T cells from 3 patients and induced exhausted cells. Data shows the average (n=20), all data was normalized to the average measured value of healthy CAR T cells (dash line). (**g**) Correlation between ratio of mitochondrial respiration to glycolysis and Piezo1 expression in induced exhausted cells over the 9-day serial CAR stimulation. Solid line shows linear regression. (**h**) Correlations of CAR T cell mechano-metabolic index and function index versus mean Piezo1 level across healthy, patients CAR T cells, and induced exhaustion through a 9-day series stimulation (D3, D6, D9). Solid line shows quadratic nonlinear fit. The mechano-metabolic index (associated with Fig. 2k) was derived by integrating multiple parameters that characterize CAR T cell mechano-metabolic function: energy, instantaneous frequency, total ATP production, glycolysis activation rate, spare respiratory capacity. CAR T cell function index was computed from geometric mean of mean Granzyme B and mean CD69 expression across all cells in respective group in h, measured by a microfluidic digital ELISA single-cell phenotyping platform. In d, I, j, m, n = 4675, 863, 2319, 1530, 706, 2403, 3489, 2657 cells for healthy (D0), D3, D6, D9 (Exhausted), patient 1, patient 2, and patient 3 group respectively. (**i**) Correlation between the functional efficacy (associated with Fig. 2k) and Piezo1-mediated Ca^2+^ influx response. Line represents linear regression. Each dot in represents the mean value from one CAR T cell group. (**j**) OCR traces of exhausted CAR T cells with or without Piezo1 blockade by GsMTx4 (n = 4) (left) or patient-derived CAR T cells with or without Yoda1 treatment (n = 3) (right), measured by Seahorse Mito Stress Assay. (**k**) Single-cell correlation of Piezo1 expression and Granzyme B production (top) and of Piezo1 expression and CD69 (bottom) and in patient and exhausted CAR T cells under Piezo1 modulation (Yoda1 or GsMTx4) n = 2381, 2492, 1021, 649 cells for patient 1, patient1 + Yoda1, exhausted, and exhausted + GsMTx4 respectively. In k, data is normalized by taking ratio of fluorescent intensity divided by background intensity of the respected group. (**l**) Quantification of transcript changes in downstream Piezo1 effectors (CAMK2D, WNT5A, RAC1) across experimental groups (n = 3). Data in j and l are shown as the mean ± s.e.m. and statistical tests used one-way ANOVA with Tukey’s post hoc test.

Having established a non-linear relationship between Piezo1 and effector output, we asked how dysregulated Piezo1 states manifest in patient-derived and exhausted CAR T cells (Fig. 5a). RT-qPCR and immunostaining analyses revealed divergent patterns: patient-derived CAR T cells exhibit heterogeneous yet lower Piezo1 expression relative to healthy controls, whereas exhausted cells displayed abnormally elevated levels upon repeated stimulation (Fig. 5b, Extended Fig 7a–c). Cellularity characterization showed that healthy donor CAR T cells were enriched in intermediate Piezo1 states with high Granzyme B, while patient-derived and exhausted CAR T cells shifted toward either low or high Piezo1 states, with corresponding loss of effector activity (Fig. 5c,d, Extended Data Fig. 7d,e). Despite the distinct Piezo1 expressions in patient and exhausted CAR T cells, both insufficient and excessive Piezo1 activity resulted in attenuated calcium responses and Rac1 activity, disrupted mechano-metabolic integration (Fig. 5e,f, Extended Fig 7f,g). Inter-patient comparisons revealed heterogeneity: Patient 3 retained stronger Piezo1-mediated calcium responses and Rac1 activation than Patients 1 or 2, correlating with superior metabolic and cytoskeletal profiles (Fig. 5d–f, Extended Data Fig. 7h).

**Extended Data Figure 8.**
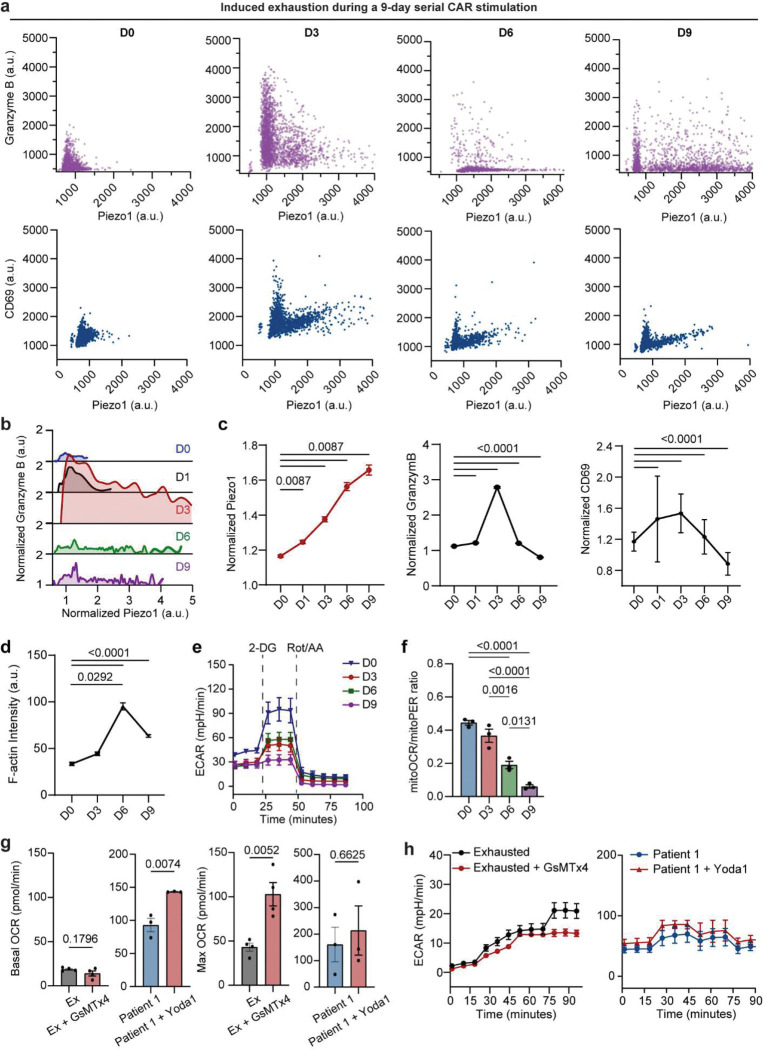
Overexpression of Piezo1 in CAR T cell exhaustion results in decline in effector and metabolic functions. (**a-b**) Microfluidic digital ELISA-based single-cell analysis of Granzyme B secretion and CD69 versus Piezo1 expression, and (**c**) time-course quantification of Piezo1 intensity, Granzyme B, and CD69 in CAR T cells during a 9-day serial stimulation (n = 4675, 863, 2319, 1530, 706 for D0, D1, D3, D6, D9 respectively). Intensity of fluorescent-stained cells was normalized to intensity of background, expressed as percentage. In b, data were binned by normalized Piezo1 expression (step 0.0038), averaged per bin, and connection line between each averaged value plotted using LOESS smoothing. (**d**) Immunofluorescent quantification of F-actin intensity in CAR T cells during the 9-day serial CAR stimulation (n = 44, 62, 156, 203 for D0, D3, D6, D9 respectively, 5 technical replicates). (**e**) Seahorse assay of extracellular acidification rate (ECAR) reveals enhanced glycolysis during induced exhaustion over 9 days (n = 3). (**f**) Ratio of mitochondrial respiration (mitoOCR) to glycolysis (glycoPER) progressively decreases with repeated stimulation over 9 days (n = 3), indicating a metabolic switch toward glycolysis. (**g**) Quantification of basal and maximal respiration and (**h**) ECAR traces of exhausted CAR T cells with or without Piezo1 blockade by GsMTx4 (n = 4) (left) or patient-derived CAR T cells with or without Yoda1 treatment (n = 3) (right), measured by Seahorse Mito Stress Assay. All data were collected from three independent experiments. Data in c-i are shown as the mean ± s.e.m. Statistical analysis in c, d and f used One-way ANOVA with Tukey’s post hoc test, in h used unpaired Student t-test.

Furthermore, we explored how Piezo1 activity changes in CAR T cells during exhaustion process, through a serial antigen stimulation with CD19^+^ leukemia cells (details see Materials and Methods). We found early-stage antigen stimulation in the first 3 days increased Piezo1 activity and effector function as evidenced by enhanced effector cytokine Granzyme B secretion and CD69 expression (Extended Data Fig. 8a–c). Notably, this Piezo1 elevation appeared to reach a functional saturation point, as further increase in Piezo1 activity induced by chronic antigen exposure impaired cytoskeletal and metabolic fitness in exhausted CAR T cells: Yoda1-stimulated calcium influx was blunted, F-actin intensity reduced, and Rac1 dynamics suppressed, indicating that Piezo1 is uncoupled from its CaMKII–NFAT/cytoskeletal effectors (Extended Data Fig. 8c,d). Metabolically, exhausted cells shifted toward glycolysis, with increased glycolytic flux and a reduced mitoOCR:glycoPER ratio, indicating impaired mitochondrial support for activation (Fig. 5g, Extended Data Fig. 8e–f).

These characterizations revealed a bell-shaped relationship, in which mechano-metabolic responses and cytotoxic function peaked at intermediate Piezo1 levels but declined at both extremes (Fig. 5h). Overall, intermediate Piezo1 as the optimal state that aligns transient Ca^2+^ transients, synaptic mechanics, and metabolic supply. While both hypoactive and hyperactive Piezo1 states drive dysfunction, Ca^2+^ flux appears to be a more reliable marker for functional outcomes of CAR T cells (Fig. 5i). This suggests that personalized profiling Piezo1-mediated Ca^2+^ signaling could stratify CAR T cell products by mechano-metabolic fitness and inform strategies for Piezo1 modulation.

As both hypoactive and hyperactive Piezo1 states are detrimental, it underscores the necessity of fine tuning Piezo1 activity to optimize CAR T cell mechano-metabolic fitness and effector function. To test whether correcting Piezo1 activity could restore function, we applied the Piezo1 inhibitor GsMTx4 to exhausted CAR T cells. Remarkably, GsMTx4 treatment rescued mitochondrial respiration, improved OCR, and enhanced granzyme B production (Fig. 5j–l, Extended Data Fig. 8g,h). Similarly, pharmacologic activation of Piezo1 with Yoda1 in patient-derived CAR T cells boosted mitochondrial function and effector activity, indicating that fine-tuning Piezo1 activity can enhance functional output across different cellular states (Fig. 5j–k, Extended Data Fig. 8g,h). Transcriptomic profiling further showed that Piezo1 modulation reprograms downstream mechanotransduction pathways, with CAMK2D, WNT5A, and RAC1 expression shifting in line with restored function, although the effect is more obvious and significant in Yoda1-treated patient cells (Fig. 5l). Together, these findings demonstrate that both inhibition of Piezo1 in exhausted cells and enhanced activation of Piezo1 in patient cells converge on restoring mechano-metabolic balance and effector capacity.

### Profiling mechano-metabolic signatures of different CAR T cell products

**Extended Data Fig. 9.**
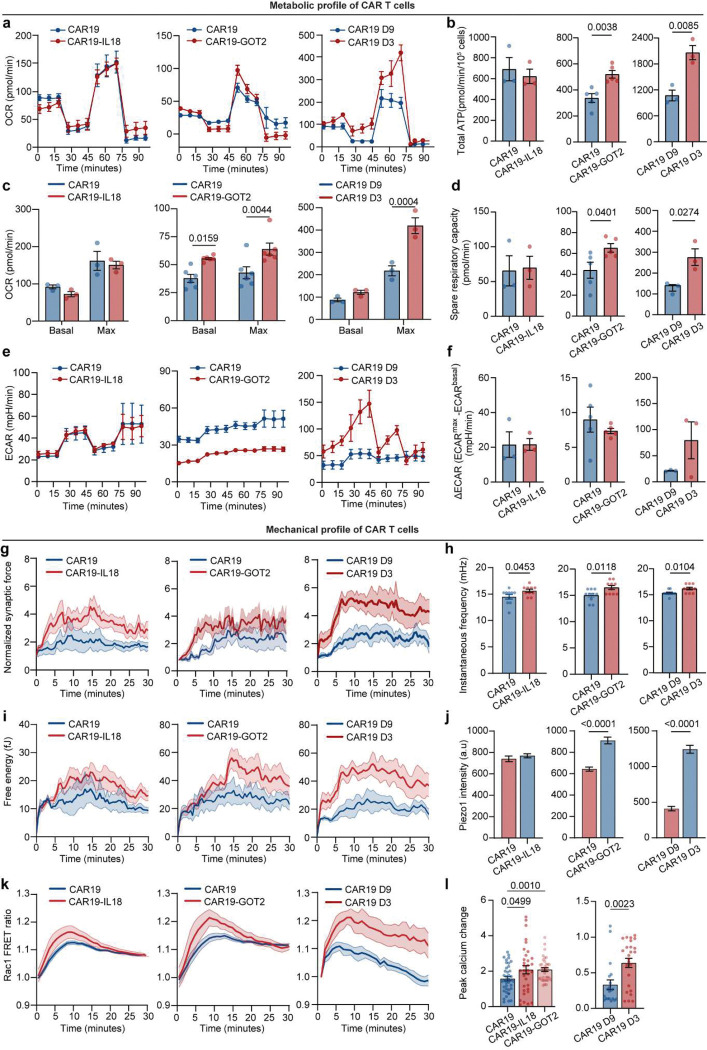
Profiling of mechano-metabolic characteristics of different CAR T cell products. **(a-f**) Metabolic and (**g-h**) mechanical profiles of various designs: 4th-generation CAR T cells expressing IL-18 (CAR19-IL18) or GOT2 (CAR19-GOT2) comparing to the 2^nd^ generation 41BBζ CAR T cells (CAR19); and 41BBζ CAR T cells generated using 3-day (CAR19 D3) vs. 9-day (CAR19 D9) manufacturing protocols. (**a**) Time-course OCR, (**b**) total ATP, (**c**) basal and maximal oxygen respiration, (**d**) spare respiratory capacity, (**e**) time-course ECAR, and (**f**) ΔECAR measured by Seahorse Mito Stress Assay across CAR T cell groups (n = 5 each for CAR19-GOT2 vs CAR19, n = 3 for CAR19-IL18 vs CAR19, and CAR19 D3 vs CAR19 D9). (**g**) Synaptic force, (**h**) force instantaneous frequency spectrum, and (**i**) free energy of 4^th^- generation CAR T cells (CAR19-IL18, CAR19-GOT2) comparing to the 2^nd^- generation 41BBζ CAR T cells (CAR19); and CAR19 D3 vs. CAR19 D9 manufacturing protocols (n = 10 cells). Data was collected from 2 independent experiments. (**j**) Piezo1 expression (n = 102, 169, 102, 40, 79 cells for CAR19, CAR19-IL18, CAR19-GOT2, CAR19 D9, CAR19 D3 CAR T cells respectively), (**k**) time-course Rac1 FRET ratio activity (n = 8 each group, 2 independent experiments), (**l**) Peak calcium influx (n = 39, 92, 36, 25, 20 cells for CAR19, CAR19-IL18, CAR19-GOT2, CAR19 D9, CAR19 D3 CAR T cells respectively) across different CAR T cell designs. Data was presented as mean and s.e.m. P-value was calculated by unpaired two-tailed Student’s t-test between two groups and one-way ANOVA with Tukey’s post hoc test among three groups.

**Extended Data Fig. 10.**
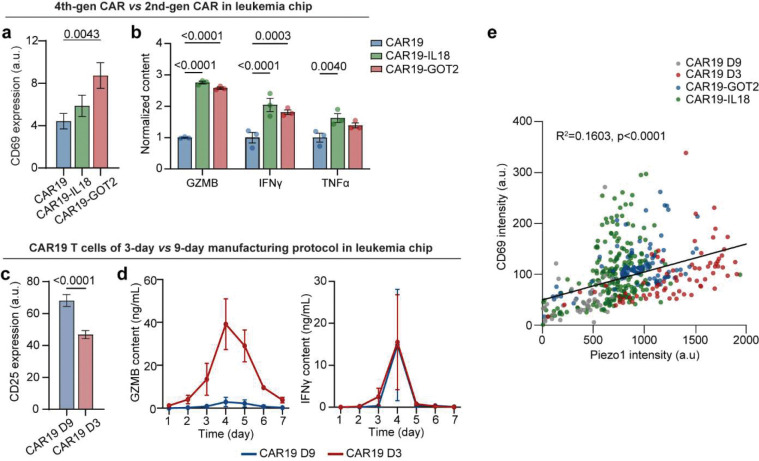
Characterizing of activations and effector functions of different CAR T cell products. (**a**) Levels of CD69 expression (n = 57 cells from 3 technical replicated.) and (**b**) effector cytokine secretion (IFNγ, TNFα, GZMB) of different 4th-gen CAR T cells after cultured 2 days on a leukemia-on-a-chip model (n = 3 technical replicates). Data in d is normalized to averaged value of CAR19. (**c**) Levels of CD25 expression of CAR19 D3 and CAR19 D9 T cells after cultured 2 days on a leukemia-on-a-chip model (n = 409 cells for D3 and 504 cells for D9 from 2 technical replicates). (**d**) ELISA measured GZMB and IFNγ secretion dynamics from CAR19 D3 and CAR19 D9 T cells on leukemia-on-a-chip for 7 days (n = 2 technical replicates, 2 independent experiments). Data in c and d was adapted with permission from ^76^. (**e**) Correlation between CD69 and Piezo1 intensity measured from immunofluorescence in single CAR T cells upon activation on a CD19/ICAM-1 functionalized glass substrates for 1 h (n = 102, 169, 102, 40, 79 cells for CAR19, CAR19-IL18, CAR19-GOT2, CAR19 D9, CAR19 D3 CAR T cells respectively). Straight lines denote the linear least-squares fits to the data. Data in b-f are shown as the mean ± s.e.m. Statistical tests in b, c used one-way ANOVA with Tukey’s post hoc test, while d used unpaired two-tailed Student’s t-test.

**Figure 6.**
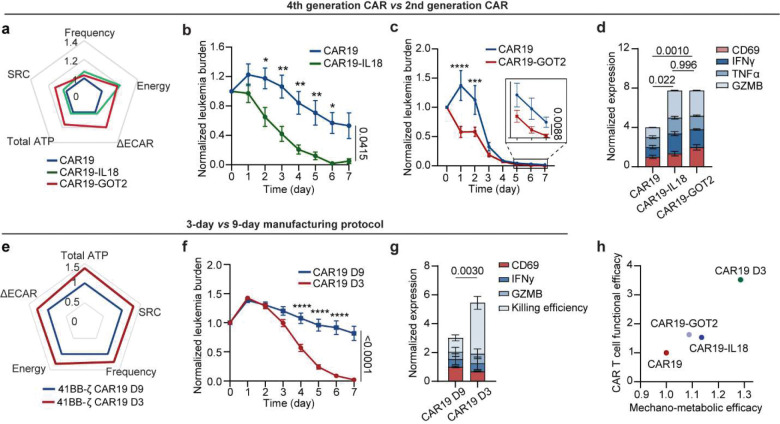
Screening mechano-metabolic phenotypes of CAR T cells of various designs and functional states. (**a**) Radar chart showing mechano-metabolic indices of 4^th^-gen CAR T cells (CAR19-IL18 and CAR19-GOT2) comparing to the 2^nd^-gen CAR19 T cells, associated with Extended Data Fig. 9. Force-related data (frequency, energy: n = 10 cells); Piezo1 expression and calcium influx: n = 20 cells. Metabolism index is the geometric mean of spare respiration capacity, total ATP, and ΔECAR. All values normalized to donor-matched CAR19 group. Data was collected from 3 independent experiments. (**b**) On-chip killing curve under treatment of 2^nd^-gen CAR19, 4^th^-gen CAR19-IL18 and (**c**) CAR19-GOT2 at 1,250 CAR T cells (n = 3). (**d**) Stacked bar chart summarizing CD69 expression (n = 56 cells from 3 technical replicates) and effector cytokine secretion (IFNγ, TNFα, GBZB) (n = 3) on different types of CAR T cells activated by interaction with leukemia cells on-chip for 2 days, associated with Extended Data Fig. 10a,b. (**e**) Representative radar chart showing the average values of mechano-metabolic profile index of CAR T cells fabricated from different manufacturing protocols (CAR19 D3 versus CAR19 D9). All data was normalized to average measured values from respected CAR19 D9 cell group, associated with Extended Data Fig. 9. (**f**) On-chip response curves under treatment of different CAR19 D3 and CAR19 D9 cells at a dose of 1,250 CAR T cells per chip (n = 4 technical replicates, 3 independent experiments). (**g**) Stacked bar chart summarizing CD25 expression (n = 307 cells for CAR19 D9, n = 392 cells for CAR19 D3 collected from 4 technical replicates), and killing efficiency (n = 4) on D3 vs D9 CAR T cells activated by interaction with leukemia on-chip for 2 days, associated with Extended Data Fig. 10c,d. Killing efficiency is absolute value of the slope of each killing curve using linear regression analysis. (**h**) Correlation between mechano-metabolic efficacy and CAR T cell functional efficacy of different CAR T products. The composite mechano-metabolic efficacy index was derived by summarizing mechanical parameters (energy, frequency) and metabolic parameters (total ATP, spare respiratory capacity (SRC), and ΔECAR) by geometric mean. Data in b, c, d, f, g shown as the mean and s.e.m. Data in f, g was adapted with permission from ^75^. Statistical comparison in b, c, f, g was performed using unpaired two-tailed Student’s t-test, and in g using one-way ANOVA with Tukey’s post hoc test. * p<0.05, ** p<0.005, *** p<0.0005, ****p<0.0001.

To validate the preclinical utility of our mechano-metabolic profiling framework in developing more effective CAR T cell therapies, we conducted a comprehensive analysis of CAR T cell products with varying designs and manufacturing protocols. We first profiled the mechanical and metabolic signatures of the 4th-generation CAR T cell products, which incorporate additional functional domains such as IL-18 or GOT2 alongside the co-stimulatory signal 4–1BB (Fig. 6, Extended Data Fig. 9). CAR19-IL18 T cells, engineered to secrete interleukin-18 (IL-18), are designed to enhance activation, proliferation, and tumor infiltration, while CAR19-GOT2 T cells, engineered to overexpress GOT2 (a metabolic enzyme) to enhance metabolic fitness and exhaustion resistance, displayed a distinctive mechano-metabolic signature. From Seahorse assays, we found that CAR19-GOT2 T cells displayed significantly higher basal and maximal OCR as well as elevated extracellular acidification compared to conventional CAR19 T cells, indicating simultaneous upregulation of oxidative phosphorylation and glycolysis (Fig. 6a, Extended Data Fig. 9a–f). This dual enhancement in energy production pathways reflects a highly metabolically fit phenotype and was consistent with their increased mechanical fitness, as demonstrated by more dynamic synaptic force generation, stronger Piezo1-mediated calcium influx, and elevated Rac1-driven cytoskeletal remodeling (Fig. 6a, Extended Data Fig. 9g–l). These combined advantages translated into superior activation and dynamic killing efficiency measured in the leukemia-on-a-chip model (Fig. 6b–d, Extended Data Fig. 10a,b), validating the enhanced in vivo performance of metabolically reprogrammed CAR T cells reported in previous studies^56–59^.

CAR T cell manufacturing typically involves T cell activation, followed by viral transduction and expansion *ex vivo* for at least 6 days. As *ex vivo* activation and expansion of CAR T cells lead to their progressive differentiation, prolonged exposure to oxidative stress, CAR T cell potency can be adversely impacted during the manufacturing process^60^. To evaluate the impact of manufacturing protocols on CAR T cell mechanical and metabolic signatures, we compared products generated using the conventional 9-day (CAR19 D9) manufacturing process with those produced via a rapid 3-day (CAR19 D3) protocol. CAR T cells manufactured using the CAR19 D3 protocol adopted a more energetical profile, with enhanced OCR and glycolytic activation (Extended Data Fig. 9a–f). CAR19 D3 cells also exhibited significantly improved mechanical properties, including higher synaptic force and calcium responses, compared to CAR19 D9 cells (Fig. 6e, Extended Data Fig. 9g–l). Functionally, CAR19 D3 cells also outperformed their CAR19 D9 counterparts in the in vitro leukemia-on-a-chip model, achieving more effective leukemia burden reduction with enhanced cytotoxicity (Fig. 6f–g, Extended Data Fig. 10c,d). These results indicate that rapid manufacturing protocols can enhance the mechanical and functional fitness of CAR T cells. Notably, across all CAR T cell products, a positive correlation was observed between Piezo1 expression and the activation marker CD69 (Extended Data Fig. 10e), suggesting that Piezo1 could serve as a potential signature of CAR T cell mechanical fitness. Moreover, the mechano-metabolic index of different types of CAR T cells strongly correlated with the functional outcomes of CAR T cells, including activation and cytotoxicity (Fig. 6h). CAR19-GOT2 and CAR19-IL18 T cells exhibited the higher mechano-metabolic index, consistent with their superior functional performance. CAR19 D3 cells had the highest mechano-metabolic index, reflecting their superior cytotoxic potential. Together, these findings demonstrate that mechano-metabolic profiling not only discriminates CAR T cell products with superior function but also provides a predictive framework for evaluating new designs and optimizing manufacturing.

## Discussion

Extensive research has advanced the biological engineering of CAR T cells by optimizing tumor antigen targeting and co-stimulatory domains to enhance cell persistence and tumor killing. However, biophysical and metabolic regulatory mechanisms remain relatively underexplored, despite being critical determinants of CAR T cell efficacy. In this study, we demonstrate that mechanical force generation and mechano-metabolic signaling are defining features of functional CAR T cells, and that dysregulation of these processes contributes to therapeutic failure. We identify Piezo1 as a central upstream regulator linking mechanical sensing to metabolic adaptation via CAMK2/Wnt/Rac1 signaling axis. Activation of Piezo1 triggered Ca^2+^ influx and downstream transcriptional and cytoskeletal programs, including Wnt3/Wnt5a ligand expression, Rac1 activation, and mTOR pathway engagement, thereby promoting glycolysis and mitochondrial respiration to fuel cytoskeletal remodeling and cytotoxicity. Conversely, Piezo1 inhibition depleted glycolytic and TCA intermediates and impaired synaptic force generation, underscoring its role as an integrator of mechanical and metabolic programs.

Unexpectedly, Piezo1 function followed a non-linear, “Goldilocks” model. Intermediate Piezo1 expression was associated with maximal Ca^2+^ signaling, Rac1 activity, and effector function, whereas both low and excessively high expression correlated with dysfunction. For instance, patient-derived CAR T cells in a hypoactive Piezo1 state exhibited low mechano-metabolic fitness and impaired effector function. Repeated stimulations caused exhaustion and a hyperactive Piezo1 state in CAR T cells. Surprisingly, this hyperactive state leads to a decoupling Piezo1 signaling from its CaMKII–NFAT/cytoskeletal effectors, where excessive Piezo1 expression no longer translates into increased Ca^2+^ influx or greater synaptic force. Instead, beyond a critical threshold, hyperactive Piezo1 initiates maladaptive signaling—potentially through chronic calcium stress, engage exhaustion-associated transcriptional programs (e.g., CaMKII–CREB–Osr2 axis^61^), or feedback inhibition of cytoskeletal remodeling—which drives synaptic dysfunction and a loss of cytotoxic potential. Metabolically, exhausted cells shifted toward glycolysis, which impaired mitochondrial support for T cell activation. These findings highlight the need to precisely tune Piezo1 activity—leveraging its benefits in acute mechanotransduction while avoiding the detrimental effects of chronic overactivation, especially in the design of CAR T cells intended for long-term persistence and activity within mechanically stressful tumor environments.

Our mechano-metabolic framework also highlights how CAR design and manufacturing conditions converge on mechano-metabolic competence as a predictor of therapeutic performance. For instance, 4th-generation CARs such as CAR19-GOT2 and CAR19-IL18 displayed enhanced mitochondrial and glycolytic capacity, greater Piezo1 activation, and stronger synaptic force generation compared with second-generation designs. Similarly, CAR T cells produced by accelerated 3-day manufacturing preserved higher Piezo1 activity and metabolic vigor than conventional 9-day products, translating into superior tumor clearance. While few strategies have focused on improving the mechanotransduction capacity of CAR T cells, our findings suggest promising future directions. For instance, co-expression of LRP6, a Wnt co-receptor known to enhance β-catenin signaling and memory preservation, has been shown to improve CAR T cell persistence and cytotoxicity^62^. Building on these insights, future engineering approaches could aim to fine-tune Piezo1 signaling in mechanosensitive-deficient CAR T cells or incorporate synthetic Piezo1-modulatory circuits into CAR constructs using synthetic biology. Altogether, targeting mechanotransduction and mechano-metabolic pathways offers a powerful route to real-time modulation of CAR T cell energy output, force generation, and persistence. This strategy has the potential to usher in a new generation of metabolically resilient, mechanically optimized CAR T cell therapies with improved efficacy in challenging tumor microenvironments.

## Materials and Methods

### Cell culture

Human anti-CD19scFv-4–1BB-CD3ζ CAR (4–1BBζ-CAR) T cells derived from healthy donors were purchased from ProMab Biotechnologies. In addition, in-house produced anti-CD19 CAR T cells with different armors (CAR-GOT2, CAR-IL18), produced with different manufactured protocols (D3 and D9 4–1BBζ-CAR19)^63^, as well as matching non-transduced T cells (NTD) were prepared with expansion for about 10 days. Leukemia patient 4–1BBζ-CAR19 T cells were obtained from the CART19 clinical trial at the University of Pennsylvania (NCT02030847). The study was approved by the Institutional Review Board at the University of Pennsylvania. It was conducted in accordance with the principles of the Declaration of Helsinki. All patients provided written informed consent. All CAR T Cells were cultured in ImmunoCult-XF T Cell Expansion Medium (Cat#10981, StemCell Technologies) supplemented with IL-2 (200 IU/mL, Cat#200–02, Peprotech) in a 37 °C, 5% CO_2_ incubator. Before experiments, frozen CAR T cells were thawed and recovered overnight at a concentration of 1×10^6^ cells/mL in 24-well plates in a 37 °C, 5% CO_2_ incubator.

To modulate Piezo1/Wnt/Rac1 signaling, CAR T cells were pre-treated with small-molecule modulators prior to functional assays. Cells were respectively incubated with 2.5 μM GsMTx4 (CAT#HY-P1410, MedChemExpress) for 30 minutes to inhibit Piezo1, 0.3 μM Yoda1 (CAT#21904, Cayman Chemical) for 30 minutes to enhance Piezo1 activity, 25 μM IWP-2 (CAT#681671–10MG, MilliporeSigma) for 24 hours to inhibit Wnt signaling, or 100 μM NSC-23766 (CAT# 8953S, R&D Systems) for 24 hours to inhibit Rac1.

To create a physiologically relevant leukemia chip model, commercial bone marrow mononuclear cells (Cat#70001.1, STEMCELL Technologies) from healthy donors were used. Human umbilical vein endothelial cells (HUVECs, Cat#C2519A, Lonza) were cultured in Endothelial Cell Growth Medium-2 BulletKit (EGM-2, Cat#CC-3162, Lonza) and used within passage 5. Primary human mesenchymal stem cells (hMSCs, Cat#PT-2501, Lonza) were cultured in Mesenchymal Stem Cell Growth Medium BulletKit (MSCGM, Cat#PT-3001 Lonza) and used within passage 5. Primary normal human lung fibroblast (NHLF, Cat#CC-2512, Lonza) were cultured in Fibroblast Growth Medium-2 BulletKit (FGM-2, Cat#CC-3132, Lonza) and used within passage 8. Osteoblasts were differentiated from hMSCs using Human Mesenchymal Stem Cell Osteogenic Differentiation Medium BulletKit (Cat#PT-3002, Lonza) for 12 days and designated as hMSC-derived osteoblasts. GFP-expressing REH B-ALL cells (Cat#Q1018798, Applied Biological Materials) were cultured in Prigow IV media (Cat#TM004, Applied Biological Materials) supplemented with 10% Fetal Bovine Serum (FBS, Cat#A5256701, Thermo Fisher Scientific) and 0.2 μg/mL Puromycin (Cat#G264, Applied Biological Materials). CD19 expressing K562-meso-19-RFP leukemia cell lines provided by Dr. Saba Ghassemi’s lab were cultured in RPMI medium (Cat#11875135, Thermo Fisher Scientific) supplemented with 10% FBS, 1% penicillin/streptomycin (Cat#15140163, Thermo Fisher Scientific), 100 μM L-glutamine (GlutaMAX Supplement, Cat#35050061, Thermo Fisher Scientific), and 50 μM β-mercaptoethanol (Cat#21985023, Thermo Fisher Scientific) in a 37°C incubator with 5% CO_2_.

### Micropillar array fabrication and functionalization

The polydimethylsiloxane (PDMS; Sylgard 184, Cat#DC2065622, Dow Corning) micropillar arrays (pillar height 5 μm, diameter 0.8 μm, effective modulus 2.557 kPa) were fabricated on a glass coverslip (22 × 22 mm, CAT#63757–01, Electron Microscopy Sciences) using a soft lithography double-molding process as previously described^64^ and in **Supplementary Fig. 1**. The glass coverslip with PDMS micropillars were then mounted onto 35 mm petri dish with a 15 mm hole in the center. Before experiments, the micropillar arrays were coated with Poly(L) Lysine (0.1 % (w/v), Cat# P8920, Sigma Aldrich) for 30 minutes at room temperature, followed by coating with protein solution containing ICAM-1 (5 ug/mL, CAT#10779–488, Peprotech) and FTIC-labeled CD19 (5 ug/mL, Cat#20–291, Acro Biosystems) overnight at 4°C. For CD8 T cell study, the micropillar arrays were functionalized with anti-CD3ε antibodies (30 μg/mL, CAT#50–123-40, Invitrogen), anti-CD28 antibodies (30 μg/mL, CAT#14–0289-82, Invitrogen), ICAM-1 (20 μg/mL), and Fibrinogen Alexa Fluor^™^ 488 conjugate (25 μg/mL, CAT#F13191, Invitrogen). Then the functionalized substrates were washed with phosphate-buffered saline (PBS, pH 7.4, CAT#10010023, Gibco) twice and with cell culture media once before cell loading.

### CAR T cell synaptic force measurement

CAR T cells stained with Calcein AM (Cat#C1430, Invitrogen) were loaded onto the functionalized micropillar arrays and immediately imaged with a Nikon CSU-X1 Spinning Disk Confocal System after cell attachment. Upon CAR T cell encountering CD19, fluorescent images of the Calcein-labeled CAR T cells and the FITC-CD19 functionalized micropillars were captured simultaneously at 30-second intervals for a duration of 30 minutes to track changes in cell morphologies and synaptic forces. The force magnitude and direction of single CAR T cells were calculated based on the displacements of the micropillars underneath cells^65, 66^. Specifically, the time series images of micropillars and CAR T cells’ cytoplasm were analyzed using open-source Cellogram software and custom-developed MATLAB programs (MathWorks) to extract cell area change, magnitude and direction of force based on displacements of the micropillars underneath the cells, as described previously^40, 67^. Mean projection of pillar displacements along the radial axis from each pillar to the cell’s center of gravity (or COG projection) were calculated to indicate the force direction, whereas outward force is positive during cell spreading and inward contraction force is negative.

### Spectrum analysis of synaptic force dynamics and Rac1 FRET activities

Deriving instantaneous frequency of synaptic force dynamics involved tracking the time series of displacement velocity for individual micropillars in response to CAR T cell activation as previously described^21^. Meanwhile, instantaneous frequency of Rac1 FRET activities in CAR T cells expressing Rac1 activity biosensors was obtained based on tracking velocity of cell edge movement^68^. To enhance the signal quality and reduce noise, Empirical Mode Decomposition (EMD) was applied, breaking down the velocity time series into a finite and small number of Intrinsic Mode Functions (IMFs). Following EMD, the Hilbert-Huang Transform (HHT) was employed on each IMF, extracting the instantaneous frequency (F(t)) and instantaneous amplitude (A(t)) at every time point (t). This process generated an instantaneous spectrum, providing both the frequency magnitude and distribution of the input synaptic force dynamics data. After comparison of the differences in 5 IMFs obtained from activated and nonactivated CAR T cells, IMF1 was chosen to calculate the instantaneous characteristics of CAR T cell synaptic force response and Rac1 activities due to its significant differences among groups.

### In vitro induction of CAR T cell exhaustion

CAR T cell exhaustion was induced through serial antigen stimulation with CD19^+^ leukemia cells over 9 days, or using anti-FMC63(CD19)/ICAM-1 antibody coated ActiveMax^®^ Streptavidin μBeads (CAT#MBS-C009–10mg, ACROBiosystems) over 8 days. To induce exhaustion with CD19^+^ leukemia cells, CAR T cells were co-cultured with K562-meso-RFP cells at a ratio of 4:1 in RPMI Medium 1640 (CAT#11875093, ThermoFihsesr Scientific) supplemented with 200 IU/mL IL-2 (CAT#200–02, PeproTech) in 24-well plates, with K562-meso-RFP cells are replenished once every three days to maintain sustained stimulation. During analysis, K562-meso-RFP signal can be sorted out according to their oversaturated red fluorescence signal. For the beads induced exhaustion, Streptavidin μBeads were first washed and resuspended in PBS, followed by conjugation with biotinylated anti-FMC63 antibodies (CAT#FM3-BY54, ACROBiosystems) and ICAM-1 (CAT#IC1-H82E8–25ug, ACROBiosystems) at a concentration of 24 μg anti-FMC63 antibodies and 16 μg ICAM-1 per mg of beads. Conjugation was performed at room temperature for 60 minutes with gentle mixing. The coated beads were then washed, collected using EasySep^™^ magnet (CAT#18000, STEMCELL Technologies), and resuspended at 2.55 mg/mL in ImmunoCult^™^-XF T Cell Expansion Medium (CAT#10981, STEMCELL Technologies) supplemented with 200 IU/mL IL-2 (CAT#200–02, PeproTech). To induce exhaustion, CAR T cells (1×10^6^ per well) were cultured in 1 mL of supplemented T cell expansion medium in 24-well plates and repeatedly stimulated with anti-FMC63/ICAM-1 μBeads at a 2:1 bead-to-cell ratio every 2 days for a total of 8 days. At each 48-hour interval, magnetic μBeads were removed using an EasySep^™^ magnet and replaced with freshly prepared anti-FMC63/ICAM-1-conjugated μBeads at the same 2:1 bead-to-cell ratio. Cell cultures were monitored daily; when the cell density exceeded 2.5×10^6^ cells/mL, cultures were split and diluted to 0.5–1×10^6^ cells/mL in fresh medium containing 30 U/mL IL-2 to maintain growth. Unless otherwise noted, exhausted cells in Figure 2–4, Extended Data Fig. 2–4 were induced via bead-based stimulation. Meanwhile, exhausted cells in single-cell cytokine assay and experiment associated with Fig. 5, Extended Data Fig. 7–8 were induced using leukemia cell–based stimulation.

### Preparation of leukemia chip for in vitro CAR T cell functional assessment

The fabrication and preparation of the leukemia chip model has been described previously^47, 69^. To construct the leukemia chip model, human leukemia blasts, bone marrow stromal cells, and bone marrow immune cells were introduced into microfluidic chips at physiologically relevant seeding densities within a fibrin hydrogel. The specific densities were as follows: HUVECs (Cat#C2519A, Lonza) at 1×107 cells/mL, hMSCs (Cat#PT-2501, Lonza) at 1×105 cells/mL, NHLF (Cat#CC-2512, Lonza) at 2×10^6^ cells/mL, hMSC-derived osteoblasts at 2×10^6^ cells/mL, GFP-expressing REH B-ALL cells (Cat#Q1018798, Applied Biological Materials) at 1×10^6^ cells/mL, and bone marrow mononuclear cells (Cat#70001.1, STEMCELL Technologies) at 5×10^6^ cells/mL. The loading process began with the injection of a 12 mg/mL sacrificial gelatin hydrogel solution (Cat#G6144–100G, Sigma) in PBS into the central area, which was then solidified at −20°C for 15 minutes to minimize bubble formation during subsequent cell loading. Next, a mixture of HUVECs, hMSCs, REH B-ALL cells, and bone marrow mononuclear cells in a fibrin solution (3 mg/mL in PBS) containing 2 U/mL thrombin (Sigma, Cat#604980–100U) was infused into the inner ring area and allowed to gel at room temperature for 10 minutes. To replicate the endosteal niche, a mixture of NHLF and hMSC-derived osteoblasts in fibrin solution (3 mg/mL in PBS) with 2 U/mL thrombin was loaded into the outer ring area using gentle vacuum suction. After gelation, cell culture media was added to the four media reservoirs, and the chip was incubated at 37°C for 30 minutes to liquefy and remove the gelatin. Finally, HUVECs were seeded in the central area to establish vascular connections with the inner ring region.

The cell culture media was formulated to support the long-term maintenance of bone marrow immune cells and tissue structure, consisting of a 1:2:1 mixture of StemSpan^™^ SFEM II (Catalog #09655, STEMCELL Technologies), Endothelial Cell Growth Medium-2 BulletKit (EGM-2, Cat#CC-3162, Lonza), and RPMI-1640 medium (Cat#11875135, Thermo Fisher Scientific), supplemented with a cytokine cocktail [Thrombopoietin (TPO, Cat#300–18, Peprotech), Interleukin-6 (IL-6, Cat#200–06, Peprotech), Interleukin-3 (IL-3, Cat#200–03, Peprotech), FMS-like tyrosine kinase 3 ligand (Flt3-L, Cat#300–19, Peprotech), and Stem cell factor (SCF, Cat#300–07, Peprotech), each at 12.5 ng/mL] and recombinant human VEGF-165/VEGFA (Cat#230–00012-10, RayBiotech) at 10 ng/mL. Fresh media was replenished daily in the media reservoirs and on top of the device.

We calculated about 10,000 REH B-ALL cells present in the leukemia after 7-day culture with an initial seeding density at 1×10^6^ cells/mL (approximately 3,000 REH B-ALL cells, day 0). After 7-day culture, the as-prepared leukemia chips were ready for assess CAR T cell functionality. We then infused 2,500 CAR T cells (ratio of effector to tumor cell = 1:4) per chip into the perfusable vessels from central sinus. We chronologically monitored and quantified the count of the GFP-expressing REH B-ALL cells daily for over 7 days to determine the CAR T killing efficiency. Culture media was collected from the chip daily for ELISA cytokine profiling. Quantification of T cell activation was conducted by immunostaining of CD69 on the leukemia chips 2 days after CAR T cell treatment.

### Immunofluorescence staining and microscopy

CAR T cells were fixed with 4% paraformaldehyde (CAT#J61899, Alfa Aesar) for 30 mins on ice and permeabilized for 10 mins using 0.1% Triton X-100 (CAT#39487, Cell Signaling Technology) (permeabilization was skipped for staining surface markers CD25 and CD69) in PBS, blocked with 3% bovine serum albumin (BSA, CAT#A7979, Sigma Aldrich) for 1 hour at room temperature. Subsequently, cells were stained with primary antibodies at 4°C overnight, washed with PBS with 0.03% Tween 20 (PBST, CAT#J77500.K2, ThermoScientific) multiple times, and then labeled with secondary antibodies for 2 hrs and DAPI (CAT#D3571, Invitrogen) for 30 minutes in 1% BSA at room temperature. The information about the antibodies and dilution conditions are listed in **Supplementary Table 2**. After staining, immunofluorescence images were captured using a Nikon CSU-X1 Spinning Disk Confocal System. For live actin imaging, CAR T cells were labeled with SPY650-FastAct^™^ (CAT#SC505, Cytoskeleton Inc.) for at 4 hours at 37°C prior to cell seeding onto the micropillar substrates. The fluorescence intensity of the staining images and F-actin z-scan distributions were quantified using the open-source software FIJI (NIH).

### Rac1 FRET imaging and analyses

Prior to FRET imaging, CAR T cells were transfected with the pTriEX-Rac1 FRET biosensor plasmid (REF) provided by Dr. Louis Hodgson’s laboratory at Albert Eistein College of using Viafect Transfection reagent (CAT#E4981, Promega) with cellular FRET/donor fluorescence emission ratio to delineate Rac1 activity patterns at the cell periphery^50^. After transfection, CAR T cells expressing Rac1 biosensors were suspended in Ham’s Nutrient Mixture F-12 Medium with no phenol red (CAT#M15350, R&D System) and plated on Poly(L) Lysine (0.1 % (w/v), Cat#P8920, Sigma Aldrich) coated glass-bottom dishes (35 mm, no. 1.5 thickness, CAT# P35GC-1.5–14-C, MatTek Corporation). Imaging was conducted at 5-second intervals for a duration of 90 minutes, using a custom microscope with 60× magnification objective lens (UIS2 60× 1.45 NA; Olympus), 2×2 camera binning on a pair of PrimeBSI-Express sCMOS cameras (Photometrics), resulting in an effective pixel size of 309 nm in the object plane. During imaging, cell illumination was achieved using Lumencor Sola white-light LED illumination through excitation bandpass filters (Chroma Technology): ET436/20X for CFP, ET500/20 for YFP, and ET480/40M for CFP, ET535/30M for YFP-FRET emissions. Post-processing of FRET images is described previously^70^.

Post-processing of biosensor FRET images followed previously established procedures^71^. Ratiometric biosensor analysis encompassed flatfield correction, background subtraction, image registration, and ratio calculations. Flatfield correction relied on a set of shading images acquired from cell-free fields of views with identical exposure and field illumination as the biosensor image sets. The donor and acceptor-FRET images were divided by shading images for flatfield correction. Background subtraction involved selecting a small region of interest in the cell-free background area, and the mean gray value from this region was subtracted from the entire field of view at each time point in the background-subtracted image sets. Subsequently, the background-subtracted images underwent image registration using a priori calibrated non-linear coordinate transformation-based approach, followed by segmentation through binary masks generated by manual histogram thresholding. The segmented acceptor-FRET channel image stacks were then divided by the segmented donor image stacks to produce the ratio data stacks. A linear pseudocolor lookup table was applied to represent relative differences in FRET/donor ratio and the corresponding Rac1 activity in cells.

### Calcium imaging

CAR T cell samples were gently washed twice with Tyrode solution (CAT#11760–05, Electron Microscopy Sciences), ensuring the removal of extraneous substances. Then CAR T cells were labeled with the calcium indicator Fluo-4 (CAT#F14201, Invitrogen^™^) at 37°C for 30 minutes, followed by washing with Tyrode solution for 3 times. To determine Ca^2+^ responses downstream of CD19-mediated activation, transfected CAR T cells were plated on anti-CD19 (2 μg/mL)-coated 35-mm glass-bottom dishes (no. 1.5 thickness, CAT#P35GC-1.5–14-C, MatTek Corporation). Imaging calcium influx was conducted at 15-second intervals, over a 30-minute period after cell attachment, using a Nikon CSU-X1 Spinning Disk Confocal System with excitation at 480 nm or 525 nm wavelength. To determine Piezo1-medaited Ca^2+^ responses, CAR T cells were plated on poly-L-lysine Poly(L) Lysine (0.1 % (w/v), CAT# P8920, Sigma Aldrich)-coated 35-mm glass-bottom dishes for 15 mins, and Yoda1 (0.3 μM, Cat#21904, Cayman Chemical) was added 2 minutes after starting recording calcium images.

### SEM imaging of CAR T cells on micropillar array

The specimens with CAR T cells attached on micropillar array substrates were washed triple with 50 mM sodium cacodylate buffer (pH 7.3; CAT#97068, Sigma-Aldrich), and were subsequently fixed for 1 hour using 2% glutaraldehyde (Cat#16010, Electron Microscopy Sciences) in 50 mM sodium cacodylate buffer. The fixation process was followed by a dehydration step, achieved by sequential immersion in 30%, 50%, 70%, 80%, and 90% ethanol (Cat#64–17-5, MiliporeSigma, then 3 immersions in 100% ethanol, each immersion lasting 10 minutes. Samples were then dried in a critical point dryer (Samdri-PVT-3D, Tousimis) following the standard protocol. The dried samples underwent a 10-second coating with gold palladium before being imaged using a Hitachi S-3400N Ultra-High Resolution SEM machine (Hitachi High Technologies America).

### Flow cytometry

After seeding on CD19/ICAM-1-coated coverslips, CAR T cell suspension was collected and gently washed with sterilized PBS and cell staining buffer (CAT#420201, BioLegend), then stained with antibodies targeting activation and exhaustion markers (listed in **Supplementary Table 2**) in flow cytometry staining buffer solutions for 15 minutes on ice. Following staining, cell samples were washed twice with cell staining buffer and resuspended in 200 μL of cell staining buffer before being subjected to analysis on an FACSAria IIIu Cell Sorter (BD Biosciences). The fluorescence intensity of each surface marker was analyzed with FlowJo 10.8.1 software. Gating strategy was established within the singlet population and was marker-specific (details see **Supplementary Fig. 4b**). Positive selection thresholds were established relative to unstained controls to ensure accurate discrimination between positive and negative populations.

### ELISA assay

Supernatant sample collected from each substrate or leukemia chip was centrifuged at 20,000 g for 15 minutes to eliminate cell debris. The concentrations of different cytokines GZMB, IFNγ, TNFα secreted by CAR T cells were measured with BioLegend’s ELISA Max^™^ Sets (CAT#DY2906–05, R&D Sytems, CAT#430207 and CAT#430101, Biolegend), following the manufacturer’s protocols using a UV-Vis spectrometer microplate reader (Sigma Aldrich).

Cytokine secretion profiles from leukemia-on-chip assays were assessed using the Human Inflammation Array C3 membrane kit (Cat#AAH-INF-3–8, RayBiotech), following the manufacturer’s instructions. Briefly, supernatants were collected from 2–4 leukemia chips at defined time points after infusion with CAR T cells or mock T cells. Samples were centrifuged at 13,000 rpm for 20 minutes at 4 °C to remove debris, then either incubated overnight with the array membranes or stored at −20 °C for later use. The membranes were then incubated with a biotinylated antibody cocktail overnight at 4 °C, washed, and further incubated with HRP-conjugated streptavidin overnight at 4 °C. A mixture of Detection Buffers C and D was applied for 2 minutes to develop chemiluminescence, which was captured using a ChemiDoc Imaging System (Bio-Rad). Spot intensities were quantified using ImageJ or Fiji with the Protein Array Analyzer plug-in (developed by Gilles Carpentier, Université Paris).

### Single cell secretion analysis with microfluidic digital ELISA

A microfluidic digital ELISA platform, adapted from the previously described PEdELISA system^72^ was used to measure single-cell secretion levels of GZMB. Briefly, GZMB-specific capture antibody was pre-coated on a functionalized glass substrate using a sandwich immunoassay format one day prior to use. Before experiments, live CAR T cells were stained with Pacific Blue^™^ anti-human CD69 Antibody (CAT#310919, Biolegend) and Piezo1 Antibody Alexa Fluor^®^ 488(CAT#NBP1–78446AF488, Novus Biologicals). The stained CAR T cells were then loaded into the functionalized device, CD69 and Piezo1 expressions were imaged on a Nikon CSU-X1 spinning disk confocal microscope. Then the device was cultured at 37 °C, with 5% CO_2_ for 4 hours to allow GZMB secretion and captured by the functionalized glass substrate. After the incubation, CAR T cells were removed using gentle PBS washing, then captured cytokine proteins were labeled with a CF^®^568 tyramide dye (CAT#92173, Biotium) through enzymatic amplification. Device was re-imaged, and pre- and post-reaction images were aligned in ImageJ (NIH) for quantitative analysis of Piezo1, CD69, and GZMB from the same single cell.

### RT-qPCR analysis

Following activation, CAR T cells were collected in RNA shield solution and lysed using the lysis buffer provided in the Quick-RNA Miniprep Plus Kit (CAT#R1057, Zymo Research), then total RNA was extracted following the manufacturer’s instructions. Reverse transcription was carried out using the RevertAid First Strand cDNA Synthesis Kit (CAT#K1621, Thermo Fisher Scientific). RT-qPCR analysis was conducted using Power SYBR^™^ Green PCR Master Mix (CAT#4367659, Thermo Fisher Scientific) on a CFX96 Touch Real-Time PCR Detection System (BioRad). The primer sequences used in the study were detailed in **Supplementary Table 3**. The expression levels of TCR activation-related genes were normalized to the housekeeping gene β-Actin, and the relative expression levels were determined using the 2^−ΔΔCt^ method.

### LC-MS metabolomics analysis

Metabolites and stable isotope incorporation were measured by LC-MS metabolomics analysis adapted from previously published approaches^73^. Samples were quenched with 1 mL pre-chilled −80°C 80:20 methanol:water (v/v). After vortexing for 1 minute, samples were returned to −80°C 30 minutes, centrifuged at 18000 × g for 10 minutes at 4°C, then supernatant was transferred to a 96-well plate and evaporated to dryness under nitrogen gas. Samples were reconstituted in 100 μL of solvent, then 2 μL aliquots (maintained at 4°C) were injected from an autosampler onto a 25 °C ZIC-pHILIC 150 × 2.1 mm 5 μm particle size column (EMD Millipore) with a ZIC-pHILIC 20 × 2.1 guard column in a Vanquish Duo UHPLC System (Thermo Fisher Scientific). Chromatography conditions were as follows: buffer A was acetonitrile; buffer B was 20 mM ammonium carbonate, 0.1% (v/v) ammonium hydroxide in water without pH adjustment. The gradient program was: 0.5 minutes at 20% buffer A then a linear gradient from 20% to 80% buffer B; 20–20.5 minutes: from 80% to 20% buffer B; 20.5–28 minutes: hold at 20% buffer B at a 0.150 mL/min flow rate. The column elute was introduced to a Q Exactive Plus with a HESI II probe operating in polarity switching mode with full scans from 70–1000 m/z with an insource fragmentation energy of 1. Instruments were controlled via XCalibur 4.1 and data was analyzed on Tracefinder 5.1 using a 5ppm window from the predominant M-H negative ion. For isotope tracing, isotopologue enrichment was calculated using FluxFix^74^.

### Total RNA-Seq

Total RNA was extracted from CAR T cell samples using a Qiagen AllPrep Mini Kit, incorporating an on-column DNaseI treatment step per the manufacturer’s guidelines. The isolated RNAs were eluted in nuclease-free water, subjected to validation for quality and quantity via UV spectrophotometry, and stored at −80 °C. The total RNA samples underwent Bioanalyzer quality control analysis using Agilent’s Bioanalyzer technology. Libraries were prepared by rRNA depletion, fragmentation, and first- and second-strand cDNA synthesis with random priming. Double-stranded cDNA was subjected to end repair, 5′ phosphorylation, 3′ adenylation, and adapter ligation, followed by PCR amplification. Final libraries were validated for fragment size and sequenced on an Illumina HiSeq platform using paired-end 150 bp reads (2×150 bp). Raw reads were trimmed with Trimmomatic v0.36 and aligned to the Homo sapiens GRCh38 genome (ENSEMBL) using STAR aligner v2.5.2b. FeatureCounts (Subread v1.5.2) was used to generate gene-level counts from uniquely mapped exon reads. Differential gene expression was analyzed using standard workflows. Gene Ontology (GO) enrichment analysis was performed on differentially expressed genes using established bioinformatics tools, identifying enriched biological processes, molecular functions, and cellular components based on adjusted p-values.

### scRNA-seq analysis

CAR T cells were oligo-tagged using TotalSeq A anti-human antibodies (BioLegend): Hashtag 5-AAGTATCGTTTCGCA (CAT#394609), Hashtag 6-GGTTGCCAGATGTCA (CAT#394611), Hashtag 7- TGTCTTTCCTGCCAG (CAT#394613), Hashtag 8- CTCCTCTGCAATTAC (CAT#394615), mixed into one suspension, and then processed on the 10x Genomics Chromium platform. The libraries were prepared using the Chromium Single Cell 3′ Reagent Kits (v3): Single Cell 3′ Library & Gel Bead Kit v3 (PN-1000075), Single Cell 3′ Chip Kit v3 (PN-1000073) and i7 Multiplex Kit (PN-120262) (10x Genomics) and following the Single Cell 3′ Reagent Kits (v3) User Guide (manual part number CG000183 Rev B). Amplified cDNA quality was assessed using the Agilent BioAnalyzer 2100 with the High Sensitivity DNA Kit, and final libraries were evaluated on the Agilent TapeStation 4200 using High Sensitivity D1000 ScreenTape. Libraries were diluted to 2 nM, pooled, and sequenced on an Illumina NovaSeq 6000 using a 100-cycle kit (28 bp Read 1, 8 bp i7 Index, 91 bp Read 2). Data processing, including quality control, filtering, dimensionality reduction, differential expression, and UMAP visualization, was performed using the Seurat package (version 4.1.1).

### Seahorse Metabolic Assay

CAR T cells were plated onto Seahorse cell culture plates coated Poly(L) Lysine (0.1 % (w/v), Cat#P8920, Sigma Aldrich) at 250,000 cells per well for 30 mins in assay media comprising Seahorse XF RPMI assay medium (CAT#103681–100, Agilent) with glucose (10 mM, CAT#103577–100, Agilent), sodium pyruvate (1 mM, CAT#103681–100, Agilent) and L-glutamine (2 mM, CAT#103579–100, Agilent), pH 7.4 at 37 °C. After cell adherence and equilibration, cell OCR and ECAR were measured during a Seahorse Mito Stress assay in Seahorse XF24 Bioanalyser (Agilent).

In Seahorse Mito Stress Assay, XF T Cell Metabolic Profiling Kit (CAT#103772–100, Agilent) was used, including addition of oligomycin A (0.5 μM), BAM15 (2.5 μM) and antimycin A and rotenone (0.5 μM each). SRC was calculated as OCR at maximum rate – OCR in basal state. Basal glycolytic ATP was calculated from ECAR prior to oligomycin; spare GlycoATP from the ECAR increase after oligomycin. Basal MitoATP was derived from OCR minus non-mitochondrial respiration (post-rotenone/antimycin A), and spare MitoATP from the Bam15-induced OCR increase. ATP values were calculated using a P/O ratio of 2.75. ΔECAR was calculated as the difference between the average ECAR post-oligomycin and the average basal ECAR.

Glycolytic activity was measured using the Seahorse XF Glycolytic Rate Assay Kit (CAT#103344–100, Agilent), with sequential injection of rotenone/antimycin A (0.5 μM each) and 2-deoxyglucose (2-DG, 50 mM). Basal proton efflux rate (glycoPER) was calculated from ECAR following subtraction of mitochondrial acidification. Basal glycolysis was calculated as the glycoPER prior to rotenone/antimycin A injection, while compensatory glycolysis was determined as the increase in glycoPER following mitochondrial inhibition.

### CAR T cell mechano-metabolic and function index calculation

To assess the mechano-energetic fitness of CAR T cells across different patient populations, we developed a Mechano-metabolic Index (MI) by computing the geometric mean of the key mechano-metabolic parameters:

$$MI={\left(E_{fe}\times f_{T}\times M_{ATP}\times M_{glyco}\times M_{SRC}\right)}^{\frac{1}{5}}\times 100$$

where $E_{fe}$ is the total energy is derived from quantified area under the curve (AUC) of free energy response curve for the first 30 min upon CAR T cell activation, $f_{T}$ is average force frequency response, $M_{ATP}$ is total ATP production from OXPHO and glycolysis, $M_{\mathrm{glyco}}$ is glycolysis activation rate, and $M_{SRC}$ is spare respiratory capacity of a cell group, and the 1/5 exponent is used to normalize across three different mechanical index to ensure that the final index has a balanced contribution from each parameter regardless of their absolute values or units. The resulting composite is to be inherently dimensionless.

To assess the functional efficacy of CAR T cells, we derived a Function Index (FI) based on their on-chip performance:

$$FI={\left(C_{L}\times A_{CD69}\times S_{Cyt}\right)}^{\frac{1}{3}}\times 100$$

where $C_{L}$ is the killing efficiency defined by absolute value of the slope of each killing curve using linear regression analysis_,_ $A_{CD69}$ represents the activation level of CAR T cells measured by CD69 fluorescent intensity after 2 days of on-chip interaction, and $S_{Cyt}$ is the cytokine secretion profile (GZMB, IFNγ, TNFα) from leukemia chip treated with CAR T cells for 2 days. The 1/3 exponent is used to normalize across three different functional parameters to ensure that the final index has a balanced contribution from each parameter.

To enable direct performance comparison across CAR T cell designs and patient-derived T cells, all indices were normalized to a reference standard. Patient-derived T cells were normalized to the Healthy control sample. CAR T cell products were normalized to 41BB, a clinically validated and widely used CAR design. Each normalized index was expressed as a percentage of its reference value, term mechanical and functional efficacy. Normalized indices were then categorized into performance tiers based on fixed thresholds: highly Functional: ≥80% of reference, intermediate: 50–70% of reference, dysfunctional: <50% of reference.

### Statistics and reproducibility

Details regarding biological and technical replicates, as well as repetitions for each experiment, can be found in the respective figure legends. All presented results, including error bars in the graphs, are shown as mean and standard deviation of the mean (s.e.m.). Representative images shown in each figure are from one of three technical replicates with similar results. To discern significant differences between two groups, an unpaired two-tailed Student’s t-test was employed with GraphPad Prism software (version 10), as specified in the figure legends. In instances involving multiple groups, comparisons were conducted through one-way analysis of variance (ANOVA) followed by Tukey’s post hoc test, also detailed in the figure legends.

## Extended Data

## Supplementary Material

Supplementary Files

This is a list of supplementary files associated with this preprint. Click to download.

• 20250929CARTSI.pdf

## Data Availability

Data supporting the results in this study are available within the paper and its Supplementary Information. The total RNA-Seq and scRNA-seq data is available in the Gene Expression Omnibus (GEO) under accession number GSE297172 and GSE306574. The raw and analyzed datasets generated during the study are available from the corresponding author on reasonable request. Source data are provided with this paper.
